# Supplementary material for: Assessment of Wild Rocket (Diplotaxis tenuifolia (L.) DC.) Germplasm Accessions by NGS Identified SSR and SNP Markers
Source: Plants (Basel). 2022 Dec 12;11(24):3482. doi: 10.3390/plants11243482 (PMC9783343; doi:10.3390/plants11243482)
Supplement: Supplementary file 1 [file plants-11-03482-s001.zip › Table S1.pdf]

Table SM1. Twenty tested SSR Loci

Locus		Sequence	Locus		Sequence
MT317459	FW	CCTTTGATCTCACCAGTC	MT317571	FW	CTTGCTGTTTGAAGTTTGC
	RV	AGCAGCAGTAATAGTGGA		RV	CGTAGCAATGACTTGCC
MT317469	FW	GCGTATGTCACCAAAGAT	MT317762	FW	CACAAGAGGCAACAGAA
	RV	CTGTGAGTAGCTGGGAA		RV	GCCTCATTCATGTTACCTC
MT317471	FW	CATTGATTGTCCCTAAGCT	MT317541	FW	GTGTGGGAGCATTTCAC
	RV	ATGCTAGGGCTCATAAGT		RV	CCTGGTGTTCTTTGCAG
MT317520	FW	CCTTAATAGCCACCTGC	MT317527	FW	ACTTTGACGAAACGAAGC
	RV	TTCTCTGCAACATCTGTG		RV	CTCAGAACCAAAGAGAAGC
MT317482	FW	CACCACGCAGACAATATG	MT317537	FW	CAGCTTTCTGTTAGTGGTC
	RV	CATATCCGTGGGTAAGGA		RV	CCAACCAAAAACGTCAGA
MT317461	FW	TGAACCTGTTAAGACTCCA	MT317610	FW	GTCTATTGATCTGATGCCG
	RV	CTCCAAAACATACCTAGACC		RV	GCGAGAACCGATCTATGA
MT317478	FW	CGATGTCTTATCCATGAGC	MT317613	FW	GTAATAAAGCGACCCTGC
	RV	TTGCAGAGTGATGGGAA		RV	CTGGTGGGAAGGTATCA
MT317577	FW	CGGATAAACATATCCGCTT	MT317734	FW	GACTAACTCTGTGTGTGC
	RV	GGTTAACATCACTAACGGT		RV	TTAATTCAGTTCCAGGCG
MT317578	FW	GCTGAACAAACCATACGA	MT317741	FW	CAAGTAATTCAGTCTCGGG
	RV	TCTAACTACGATCAGGACAG		RV	GGTAAAGCAGGGTCTTG
MT317582	FW	GCAAACAAAGCCAATCC	MT317823	FW	CCAACATAGAAAGGTGCG
	RV	CTTCTCGTCTTCTTCAGGA		RV	GAGTTCCTCCAAAAGCTG
