# Supplementary material for: Assessment of Wild Rocket (Diplotaxis tenuifolia (L.) DC.) Germplasm Accessions by NGS Identified SSR and SNP Markers
Source: Plants (Basel). 2022 Dec 12;11(24):3482. doi: 10.3390/plants11243482 (PMC9783343; doi:10.3390/plants11243482)
Supplement: Supplementary file 1 [file plants-11-03482-s001.zip › Table S2.pdf]

Table SM2 . Sequences of the analyzed SSR and SNP loci. The sequences used for primer design are underscored and highlighted in bold. The microsatellite sequences are in italic.

SSR *Loci*

MT317577

TTAGAAAAAAAATAACGATATTTTAGATAAAATATTGAGTATTTTGAAAATTATCAAATTGT
TAGAATGATTTAGATAAAAAGAGTTCAGATAGATAGGATTGTTTTGGATATTT**CGGATAAA**
CATATCCGCTTACTTTCAGGATTGATGATTGATAATTTTTTAAAATATTTTCGAAGACTTTT
AATAGATTTTTAAAT*TATATATATATATATATATATATATA*TTTGGCTATGCTATGTATATAATTAAT
ATTTGGGTTTTTAGTGATTTAAACTCCCAACTATTTTTAAA*TCGGAAGAAAAAC(C)TCC*AACTA
AAATTTTATGTTTTTAAACCTTGAACTATTAA**ACCGTTAGTGATGTTAACC**TTCCGTTAAAA
AATTCGTTTTTCCTAAACAGACATCCGTTAAATTGAAACGCGGTGTTTCATATAATCACATAA
AGGACACGCAATGTTTATAATCAACAAATATTTTAGTGATTTAAACCTCTAACTATTTTTAAA
TAGGATG

MT317527

TGATGAACAAAAAAAATTCACCATAATTTTTTTCGTAAACATGGTGTAGCAGTCGTTGTTAA
TTGCCTTTACGAAAGATACTTTATTTCCACGGTTTATGAAATGACCAGATAAGTCCGATATAT
ATAGTCGGATCAAACATTTTAGAATATTTCTATTTACATGATCTGTACAACATAGTCAGGAA
TGGAGAAGGAAAATGAGAATAAATCCCAACTGCTATTATCTGATATGAGATAATTAGTCACT
AAGATCATATCAGAGGGTCAACCCGCGTG**ACTTTGACGAAACGAAGC**CC*GTGTGTGTGTGT*
*GTGTGTGTGTGT*GAGTGAAGCTGGTTAGGGTTTTGTCAGAAGGCTCGACAGAGTGTATTAGTC
GTGGTCAAGTTAGAAGAGTGTTGCTGTTCAATGGGGCTGCTGCTTGTGGGACTACAGATTCT
GCTTCTCTTTGGTTCTGAGATCCTCACGTTGGAGATTGCAACCACTAGCACATCTACCACA
CCGCTCTCATCTTCTT

MT317537

GATGAAGGTTCCGAGAACTAACCAGCTTGGTGTCAAAACCAAAGATGGGTTATACTATAAA
TTCACTGGCTTTCGTGACCAGGTTTGTGTTTTGCAGACAAGATTCCTTTGTCTTTGCTTGAAG
CTTATGTGTGTGTTCTTTTGGTGGTGTAATAGGATGTTGCGAGCCTGACAAGTTTTTTCCAAA
GTGCGTTTGGGAAAACACCTGAAGAGAAA**CAGCTTTCTGTTAGTGGTC**GGAATTGGGGAG
AAGTGGATTTGAATG*GTGTGTGTGTGTGTGTGTGTGTGTTGT*CCTTCGCATTTAAGCTTCTTATGT
GGATAAAGGTCTTAGCTTTATTTCTTTTTTGTTTGTGTGTGTGTGAAGGGAATAA**TCTGACGT**
TTTTGGTTGGGGGAAAGCAAGCTTTTGAGGTGTCTCTGGCTGATGTTTCACAGACTCAGCTT
CAAGGGAAAAATGATGTTTTGTTGGAGTTTCATGTTGATGACACTGCTGGTGCCAATGAGGT
ATGATATTGTATGT

MT317610

TTAGTGATTATGCTAGGTTGAAACGGTTTTAAAAGTAAATTGCAGGTTTGTGAGAAATGGTA
ATTTGCTCGATTGAATCGGTTTTGGTATTAAACAATAAGGAAAGGTAGTTAGATTTAAGATT
ACTATTCAGATAATCAGGATTATAATGCTATATATGCCTAATAAGTTGCATGCATGATATTA
AAAAGATTGAAATATGCTAATATGTCCTCGCGTTTTAGACTC**GTCTATTGATCTGATGCCG**
AAAATATCGATCGATGTTTTGACAGCGATATCGA*TATATATATATATATATATATA*TTTGTCGTC
GATCGGTTACTCTATACGAGTATCGATCGATATAGCTCAGATCGAGTTGATGTGGATGCTCA
CCAAGA**TCATAGATCGGTTCTCGC**GTATCCCTAGAGGATCTTAGCGTATGCAAGATGATGT
TAGGATGATGATTAACGAGTTAATTCATTCCAAGATCATATTCTAGTTTGCTACTCTAGGATA
AACATTAGAGTTCA

MT317823

CTCTTATTGCACCTTTCACGCCCTGAATATTAACACAATGATGTTAAAGATCCTTCCACTTAT
CACTCAGATGAAAACAAAATGCTAAAACATAACCTTAAGTGGAAATCCCAAGCATATTACA
GCTGAGACTCTGATATCTTCACTCACGGCTACCATGCAACTTACTCTGCAGAAGCATAAGAT
CACAT**CCAACATAGAAAGGTGCG**TAACAGAACAACGTGC*GAGAGAGAGAGAGAGAGAGAGA*
*GAGAGAGAGA*TCTACCTGGAACCCATTGATTTGCCAGCCAGTATCAAAGGATGACCAGGGAA
TCTAGCAGCAGTTTCTTTCACAAAATCCAGATGAAATTCAGTAAGTTTTT**CAGCTTTTGGAG**
GAACTCTTCTTTTCCCTCCAACAATATCTACCCACCCAGAAATTTCCACAAATTAAATTGAA

TCCCCAATAAGTTTCTATCAAGAACACGAATATTATTATTAAAAAAAAATCTAGAATTTTCA
CAGACATGGGTAATCAA

SNP *Loci*

DiploTSNP.045

TCTTTTCACTCGAGAATGATCCATTTGTTAGTAGACTTTCTATTAACCATTATAAATATTGTA
TATAATGTCTTCTTTTGAGCACA**GTCCCATGATTAGATATGGT**TGTATATGTCTAATTAATT
TTTCTTCTCCATCGTCTCAGTGTAATAGATATGCTTGCCTTACTTGGTGGAAATATGAATGGA
CTATGGTTCCTGAATGTTCATTTCCGAGCCTACAAACTTTC**(*G/T*)**AAAGGAAGTTATACTTTG
GAGAACCAAAAGAAAGAGATCTCGTGGTTTATATCTTGGAAAA**CGCTTGTCACTTAAAGA
CT**GTAACAATCTCGGTGAGAGACTGTAATGATGTTCAAAAACTTAAGATGATCAAAGAGTT
GGCACTTTCTTCTCGAACTTCAACTGCATGTCAACTCATATTCGAATAATCTTATGTTTATAT
TTTTATGATTTGAACTTTGCTTCTATATGTATCCGA

DiploTSNP.062

TACTTTATCAAAAACAGAAAATGTTTGGGAAAATACATGTGAGTTTTTTCGAAAGACATTCA
ATATAAGGAAACATTATAATAGACCATGTAACTTATCTCAAATTTTTTTTCTTTGGTTCTATA
T**GCGTTTGAATGGTTTCTG**GAAATTTTAGGTATAACCATGACTATGTTGGTTTATAGGATTA
TGTCTAAGTGATG**(*T/C*)**CGAAAAAAGAAGGAGCCTTACTGAAAATCAAATAATTTTTGAGAA
GTAATGGAACTTCATTAGATAATTGGACATCAATGCTTAAAGCCTTCTGCAACTTTAATGAC
TCTAAAAATGTTTTGATCATGAATGAGCTGAGTTATAACCAAAAAGAGTTTAGGGAAGAAA
ATGATAGAGACATCTTAAAA**TTGACGGATGAGCAAAG**GAAGATATACAAAGAAATTATTG
ATGATGTTTTAGAAAAAGAAGGGTGTTATTTTCGTTTAT

DiploTSNP.090

TTGATGAATGATCAAAACGTATTTTATTGAGAGAAGTAACATACAAACATGCGTATAGAACT
GACTCTTAACGAAGACTATCATCTGTACGTGTTCCTGGGACATCTTTCATGTCACTGTCACGG
TG**CATCTGTGCCTGATCTC**CAGTGATTTCAGACACTGCCTGTTCCTTGCCCTTTTC***(G/T)***ATA
CCTTGAGCGCTTGAGAAGAGCTCTACAGGCTCAGACTCTGCGATCTTGCCTCCTTCAAGTGG
TGTCCCATGTTTGTATCTCACTCGTATAGCCTCGTCTTCAGACTGCTTCGCTTGAGCTGTTGC
GTAGTTTATGTCGTCTTTGTTCTTTGCCATCTTTAGGGTTTAA**TGAATCACGACTTGACATG**
GGACCGTATATAAACAGAGTTAGGTTCTTCGTGTCACGCTCTGGACTCTCGTGTTGTGCTTAC
TTCTCTTTGTTGTGTGTTCTCTTTTGTTGACAC

DiploTSNP.138

AAATAGCATTTACTCTCTTTAACCTAGTACCTTCGATGTACACATTATATAAATACTAGTTGT
ATTGTCTTCTTCAATAATAAGAAACACATTACATTTACATGGTATCAA**GAGCCTTGTAACAG
ATCC**AAAATTTTCTTTCTCTCCTTTCTTCTCACTACACACCAACTCCATTCATCATGTCTACGA
CCTCTGCTGAAACAG***(G/T)***CGACACAAATCCACAAACCCTCCTCAATCTGAACATGACAAAC
GTCACGAAACTAACCTCCAGCAACTACATGATGTGGAGCCTCCAAGTTCAAGCTCTCCTTGA
CGGCTATGATCTCAGCAGCCACCTTGACAACTCCACTCCACCACCGCCACCAACCATAACCA
CTGACGGAA**CAACAGTCACAAACCCAG**AGTTCGCCCTCTGGAAACGTCAAGACCGGCTAA
TCTTTAGCGGTCTCATCGGAGCCATCTCAGTTCCCA

DiploTSNP.213

GAATCCCCTACTGTCTAAGCACCGTCTACTTCTCATTTCCATTTGAACAGCAAATTGGACAAT
ACTTAACCTGAATTCACCGCGAGA**TGGCATCCTACTCTTTCTC**GGCCTACCTGGTGGGCGG
CGTGTTGCTGGCGGGAAGAGCAACCCATTGTCTCCTTCCACACCCATCTTAATTGATGTTATG
TTGGTGTGGTCAACGGGATTGATGCTTCCACTGTAAGCCATTGCCCAGTATGCGGTGCTGTA
AACTGGGT(***T/C***)GACCTTACTCTCCACAGACTCTTTAGCATAAATGCCCGCAG**CCATATCAT
GAACACACGG**TATGGCTAGCGCTTCAAACTCCTTGCAAGACCATTTTTTGTTCACCAGATCG
ACTCTTGAATACATTCCATTTTCATCCACAACCTCGTACTCTCCCTCTATGATGTGGTGGACA
CCATATTCTGAACCCTCGCTAAAGTTTTTTGCCAA

DiploTSNP.220

AAATGTACGCCGTGCAATGCAAGATATTGATCTTGGTAGCAATGATGCTCCTTTTGTGCTAC
CTGTTGATGTGGTGAATCGGGCTGCTGAAGAAAATATATTCATTCTGGTGGGGAGTCCAACG

ATGCCACGGAAACAGAATCTCAGAGGCATCATC**TCTACGATGCCAAGAGTT**TGGGGGTTA
GAAGGGATTGTTCGTGGGCAAATTATGGAAGGAAGAAGATTCCAATTCATCTTTCCAAGTGA
GGAAT**(*T/C*)**GATGGACAACGTACTCAGACGTGGTCCGTGGGCTTATGCAGATAGGATGATCA
CGCTTCAGAAGTGGACTCCGTTGATGGATTTGGCTTTGTTGAACTTCATTCCTTTTTGGATTC
AAGTTCGT**GGGATACCTTTCCAGTACA**TGAATCGTTAAGTGGTGATCAATATTGCAAGATT
GATGGGGCAATACATCCAAGTGGATTACAATGAAGAACTTGTAGGGCGTTTGGAGTTTGTTC
GTATCAGACTTAATTGG

DiploTSNP.270

AAATAGGTGGCTTATCAGTTTTTCACACTCCACTTGTGTCAGGCACACATTATAAGCATCGA
GTCACATGCCCTGACTCAGTT**CTAATGAGTTCGTCGTTCG**CCATTAGCTTTTTACATCTATA
AATATATTAAGTGACATCTCAATGTTTATTGCAAACCTAGTTTTTTTTTTCATAAGGCTTCTTT
TC**(*A/G*)**AGTAGTTTGCACAAAGTAGAGAGTGAATAAAATATAATAGAATGTATGCATTAGTA
GAATTTGACTTGGTTCTCCATTACAATGGCCACGCTGCATGTGCAATATCTTTCAAGAACGA
AGGTGATGATGACGATGATGTAGTCTATGATTACGCTCCAGCTGCATGAGTCAATCATCTTT
AGTTATGACGTCTTCAC**CACCAGGAAGAGATAAGCT**TTTCTAGCTAGCTAGATACAACCCT
AATTAGCAATATATGTATTTTTAGCAATATATGTAT

DiploTSNP.290

CCACCTTAATGTGTGCTGAGCTGGATTAGTGTAAAGATGAGTGATATATCAAAATTAACTAG
TGAAAATGTG**CGAAAGAGATGCTATCGG**CTTTCATGAACCTTATTGTGTAATCAATCATAA
AAGGCTTTACTGCACAATGTATACAAATTAGAAAGCTGATGAGAAGTAAACAATTTGCAAT
ACAAACTAAAGAAACTGGTAAAGAAAAATAGTGGTCTTTGATATAGGAATCTACG**(*T/C*)**CGA
TTATGTGCCCATCATATCCCCATCTGTTAATAACTTCAACACATCAGCTCATC**GGTTAGAAC**
TGAGGACGTACCATCCTCGTCTTTTTTTATCATTTGCAATTGCATTGTTACCGGCACATCAGC
TCATCGGTTGTTCACTGATCCGAAATTATTATATACTGTACTACTTTTTTAAAATGTCGGCAA
GGAAATAGTCATCTGAATTATTTTATGGTTCAGAACT

DiploTSNP.448

GGTTTCTCTATAGTGCGGAGGGGTTTTGGATGGTTGCTCGACCTCATGAAATGCCGATCTGG
AGGTTGATCTGGTTCTCGAGTTT**GGCTGGATATGTGTCAGA**TCTACTTCGGAGAGTTTTCCA
AAATGTCAAAGTGTCACTTCCTTTTCATTTCTGGATAGCTACACTGCATCCTCTTGTCTCCAT
CTCTCTCATTTTTCGGTTTTTTCGT**(*A/C*)**GAGGTTTGAGGCTTTCATCAGGAACATCTTTGCTT
ATCATTCTTGCAATAATTTAGCATGACGATTCTGCATGATTGGACTTCCTTTCAACTACCTCG
GCTGTTTTTGGAGCTTTTGCCTCCTCTAATTTTTATA**GTTCCAGAAGGAACATGAC**TGATGT
TTTACTTTGCATTTTTCTATTTTCTGAAGCTGTAGTCATTTATGAATGTTTTTCTCGAGTGAAG
GGACCGATGTTGAGTGCTTAAAACCTCTTCA
