# Supplementary material for: Assessment of Wild Rocket (Diplotaxis tenuifolia (L.) DC.) Germplasm Accessions by NGS Identified SSR and SNP Markers
Source: Plants (Basel). 2022 Dec 12;11(24):3482. doi: 10.3390/plants11243482 (PMC9783343; doi:10.3390/plants11243482)
Supplement: Supplementary file 1 [file Table S3.pdf › Table S3.pdf]

Table SM3 Genetic similarity matrix (SSR markers)

Accessions:	1	3	4	5	6	7	8	9	10	12	15	16	17	18	19	20	21	22	23	24	26	27	29	32	35	36	37	38	39	40	41	42	43	44	45	46	47	48	49	51	52	54	55	56	57	58	59	60	61	63	64	65	68	69	72	73	74	81	84	91	92	97	98	99	100	101	102	103	104	105	134	146	152	154	155	156	157	158	159	160	161	162	163	164	165	166	167	168	169	170
**1**	1.000																																																																																												
**3**	1.000	1.000																																																																																											
**4**	0.900	0.900	1.000																																																																																										
**5**	0.600	0.600	0.700	1.000																																																																																								
**6**	0.600	0.600	0.700	0.800	1.000																																																																																							
**7**	0.700	0.700	0.800	0.800	0.900	1.000																																																																																					
**8**	0.500	0.500	0.500	0.500	0.500	0.500	1.000																																																																																		
**9**	0.700	0.700	0.800	0.600	0.700	0.800	0.500	1.000																																																																																
**10**	0.600	0.600	0.500	0.600	0.500	0.500	0.700	0.500	1.000																																																																															
**12**	0.700	0.700	0.800	0.700	0.600	0.700	0.400	0.800	0.400	1.000																																																																													
**15**	1.000	1.000	0.900	0.600	0.600	0.700	0.500	0.700	0.600	0.700	1.000																																																																											
**16**	0.600	0.600	0.500	0.600	0.500	0.500	0.700	0.300	0.800	0.300	0.600	1.000																																																																										
**17**	0.400	0.400	0.400	0.500	0.500	0.500	0.900	0.500	0.600	0.400	0.400	0.600	1.000																																																																									
**18**	0.600	0.600	0.500	0.500	0.500	0.500	0.700	0.300	0.700	0.300	0.600	0.900	0.600	1.000																																																																								
**19**	0.600	0.600	0.500	0.600	0.500	0.500	0.700	0.500	1.000	0.400	0.600	0.800	0.600	0.700	1.000																																																																								
**20**	0.800	0.800	0.900	0.600	0.600	0.700	0.500	0.700	0.400	0.700	0.800	0.500	0.400	0.500	0.400	1.000																																																																						
**21**	0.400	0.400	0.500	0.600	0.500	0.500	0.500	0.600	0.500	0.500	0.400	0.400	0.500	0.300	0.500	0.500	1.000																																																																					
**22**	0.400	0.400	0.400	0.500	0.400	0.400	0.500	0.500	0.500	0.400	0.400	0.400	0.500	0.300	0.500	0.400	0.900	1.000																																																																				
**23**	0.300	0.300	0.300	0.500	0.400	0.400	0.500	0.400	0.600	0.300	0.300	0.500	0.500	0.400	0.600	0.300	0.600	0.700	1.000																																																																			
**24**	0.400	0.400	0.400	0.500	0.500	0.500	0.900	0.500	0.600	0.400	0.400	0.600	1.000	0.600	0.600	0.400	0.500	0.500	0.500	1.000																																																																		
**26**	0.500	0.500	0.600	0.600	0.700	0.700	0.300	0.600	0.400	0.500	0.500	0.400	0.300	0.400	0.400	0.500	0.400	0.300	0.300	0.300	1.000																																																																	
**27**	0.500	0.500	0.600	0.700	0.900	0.800	0.600	0.800	0.600	0.600	0.500	0.400	0.600	0.400	0.600	0.500	0.600	0.500	0.500	0.600	0.600	1.000																																																															
**29**	0.118	0.118	0.118	0.235	0.235	0.235	0.353	0.235	0.353	0.118	0.118	0.235	0.353	0.235	0.353	0.118	0.235	0.235	0.353	0.353	0.118	0.353	1.000																																																													
**32**	0.700	0.700	0.800	0.800	0.700	0.700	0.500	0.600	0.600	0.600	0.700	0.600	0.400	0.500	0.600	0.800	0.600	0.500	0.400	0.400	0.600	0.600	0.118	1.000																																																												
**35**	0.800	0.800	0.700	0.600	0.500	0.500	0.500	0.500	0.600	0.500	0.800	0.700	0.400	0.600	0.600	0.700	0.500	0.500	0.400	0.400	0.400	0.400	0.118	0.700	1.000																																																											
**36**	0.800	0.800	0.700	0.500	0.600	0.500	0.500	0.500	0.500	0.500	0.800	0.600	0.400	0.600	0.500	0.700	0.500	0.500	0.300	0.400	0.400	0.500	0.118	0.600	0.800	1.000																																																									
**37**	0.900	0.900	1.000	0.700	0.700	0.800	0.500	0.800	0.500	0.800	0.900	0.500	0.400	0.500	0.500	0.900	0.500	0.400	0.300	0.400	0.600	0.600	0.118	0.800	0.700	0.700	1.000																																																							
**38**	0.300	0.300	0.300	0.500	0.400	0.400	0.500	0.400	0.600	0.300	0.300	0.500	0.500	0.400	0.600	0.300	0.600	0.700	1.000	0.500	0.300	0.500	0.353	0.400	0.400	0.300	0.300	1.000																																																						
**39**	0.400	0.400	0.400	0.500	0.400	0.400	0.500	0.500	0.500	0.400	0.400	0.400	0.500	0.300	0.500	0.400	0.900	1.000	0.700	0.500	0.300	0.500	0.235	0.500	0.500	0.500	0.400	0.700	1.000																																																					
**40**	0.600	0.600	0.600	0.500	0.500	0.600	0.700	0.700	0.500	0.600	0.600	0.400	0.800	0.400	0.500	0.600	0.500	0.500	0.500	0.800	0.300	0.600	0.353	0.400	0.500	0.500	0.600	0.500	0.500	1.000																																																				
**41**	0.700	0.700	0.800	0.700	0.800	0.900	0.500	0.800	0.400	0.700	0.700	0.500	0.500	0.500	0.400	0.800	0.500	0.400	0.400	0.500	0.600	0.700	0.235	0.600	0.600	0.600	0.800	0.400	0.400	0.700	1.000																																																			
**42**	0.700	0.700	0.700	0.800	0.700	0.700	0.600	0.500	0.700	0.500	0.700	0.700	0.500	0.600	0.700	0.600	0.500	0.500	0.500	0.500	0.500	0.600	0.235	0.800	0.700	0.600	0.700	0.500	0.500	0.500	0.600	1.000																																																
**43**	0.600	0.600	0.700	0.800	0.900	0.800	0.500	0.600	0.500	0.600	0.600	0.500	0.500	0.500	0.500	0.600	0.500	0.400	0.400	0.500	0.600	0.800	0.235	0.700	0.500	0.600	0.700	0.400	0.400	0.500	0.700	0.800	1.000																																																	
**44**	0.600	0.600	0.600	0.600	0.700	0.600	0.600	0.500	0.600	0.600	0.600	0.500	0.500	0.500	0.600	0.500	0.400	0.400	0.400	0.500	0.400	0.700	0.235	0.600	0.500	0.600	0.600	0.400	0.400	0.500	0.500	0.700	0.700	1.000																																																
**45**	0.800	0.800	0.800	0.700	0.700	0.800	0.600	0.600	0.600	0.600	0.800	0.600	0.500	0.600	0.600	0.700	0.400	0.400	0.400	0.500	0.500	0.600	0.235	0.700	0.600	0.600	0.800	0.400	0.400	0.600	0.700	0.900	0.800	0.700	1.000																																														
**46**	0.600	0.600	0.700	0.600	0.700	0.800	0.600	0.900	0.500	0.700	0.600	0.400	0.600	0.400	0.500	0.700	0.600	0.500	0.500	0.600	0.500	0.800	0.353	0.500	0.500	0.500	0.700	0.500	0.500	0.800	0.900	0.500	0.600	0.500	0.600	1.000																																												
**47**	0.600	0.600	0.700	0.600	0.600	0.700	0.500	0.800	0.500	0.700	0.600	0.300	0.500	0.300	0.500	0.600	0.600	0.500	0.400	0.500	0.500	0.700	0.235	0.600	0.400	0.400	0.700	0.400	0.500	0.600	0.600	0.600	0.700	0.500	0.700	0.700	1.000																																										
**48**	0.700	0.700	0.800	0.800	0.800	0.800	0.400	0.800	0.500	0.700	0.700	0.500	0.400	0.400	0.500	0.700	0.600	0.500	0.400	0.400	0.700	0.700	0.118	0.800	0.700	0.600	0.800	0.400	0.500	0.500	0.800	0.700	0.700	0.500	0.600	0.700	0.600	1.000																																										
**49**	0.600	0.600	0.700	0.800	0.700	0.700	0.500	0.600	0.500	0.600	0.600	0.600	0.500	0.500	0.500	0.700	0.600	0.500	0.500	0.500	0.500	0.600	0.235	0.700	0.700	0.600	0.700	0.500	0.500	0.600	0.800	0.800	0.800	0.500	0.700	0.700	0.600	0.800	1.000																																									
**51**	0.700	0.700	0.800	0.700	0.600	0.700	0.400	0.800	0.400	1.000	0.700	0.300	0.400	0.300	0.400	0.700	0.500	0.400	0.300	0.400	0.500	0.600	0.118	0.600	0.500	0.500	0.800	0.300	0.400	0.600	0.700	0.500	0.600	0.600	0.600	0.700	0.700	0.700	0.600	1.000																																								
**52**	0.500	0.500	0.600	0.700	0.700	0.700	0.300	0.600	0.500	0.500	0.500	0.500	0.300	0.400	0.500	0.500	0.500	0.400	0.500	0.300	0.800	0.600	0.118	0.700	0.500	0.400	0.600	0.500	0.400	0.300	0.600	0.600	0.600	0.400	0.500	0.500	0.500	0.800	0.600	0.500	1.000																																							
**54**	0.500	0.500	0.600	0.600	0.600	0.600	0.400	0.600	0.400	0.600	0.500	0.300	0.400	0.300	0.400	0.500	0.500	0.400	0.300	0.400	0.600	0.600	0.118	0.600	0.400	0.400	0.600	0.300	0.400	0.400	0.500	0.600	0.700	0.500	0.600	0.500	0.800	0.600	0.600	0.600	0.500	1.000																																						
**55**	0.400	0.400	0.500	0.500	0.500	0.500	0.500	0.500	0.400	0.500	0.400	0.400	0.500	0.400	0.400	0.600	0.600	0.500	0.400	0.500	0.400	0.500	0.118	0.600	0.400	0.400	0.500	0.400	0.500	0.400	0.500	0.400	0.500	0.400	0.400	0.500	0.600	0.500	0.500	0.500	0.400	0.700	1.000																																					
**56**	0.556	0.556	0.556	0.444	0.444	0.444	0.556	0.667	0.444	0.556	0.556	0.333	0.556	0.333	0.444	0.556	0.556	0.556	0.556	0.556	0.333	0.556	0.267	0.444	0.667	0.556	0.556	0.556	0.556	0.667	0.556	0.444	0.444	0.444	0.444	0.667	0.556	0.556	0.556	0.556	0.444	0.444	0.444	1.000																																				
**57**	0.500	0.500	0.600	0.600	0.600	0.700	0.700	0.700	0.500	0.600	0.500	0.400	0.700	0.400	0.500	0.600	0.600	0.500	0.500	0.700	0.400	0.700	0.353	0.500	0.400	0.400	0.600	0.500	0.500	0.800	0.700	0.500	0.600	0.500	0.600	0.800	0.800	0.500	0.600	0.600	0.400	0.600	0.600	0.556	1.000																																			
**58**	0.600	0.600	0.700	0.700	0.600	0.600	0.500	0.800	0.600	0.700	0.600	0.400	0.500	0.300	0.600	0.600	0.700	0.600	0.500	0.500	0.500	0.700	0.235	0.700	0.600	0.500	0.700	0.500	0.600	0.600	0.600	0.700	0.700	0.500	0.600	0.700	0.800	0.800	0.800	0.700	0.600	0.700	0.500	0.667	0.600	1.000																																		
**59**	0.500	0.500	0.600	0.900	0.700	0.700	0.600	0.700	0.700	0.700	0.500	0.500	0.600	0.400	0.700	0.500	0.700	0.600	0.600	0.600	0.500	0.800	0.353	0.700	0.500	0.400	0.600	0.600	0.600	0.600	0.600	0.700	0.700	0.600	0.600	0.700	0.700	0.700	0.700	0.700	0.600	0.600	0.500	0.556	0.700	0.800	1.000																																																		
**60**	0.400	0.400	0.500	0.700	0.700	0.700	0.600	0.700	0.600	0.500	0.400	0.500	0.600	0.400	0.600	0.500	0.800	0.700	0.600	0.600	0.500	0.800	0.353	0.600	0.500	0.500	0.500	0.600	0.700	0.600	0.700	0.600	0.600	0.500	0.500	0.800	0.600	0.700	0.700	0.500	0.600	0.500	0.500	0.556	0.700	0.700	0.800	1.000																																																
**61**	0.500	0.500	0.600	0.800	0.700	0.700	0.600	0.700	0.700	0.600	0.500	0.500	0.600	0.400	0.700	0.500	0.700	0.600	0.600	0.600	0.500	0.800	0.353	0.700	0.500	0.400	0.600	0.600	0.600	0.600	0.600	0.800	0.800	0.600	0.700	0.700	0.800	0.700	0.800	0.600	0.600	0.700	0.500	0.556	0.700	0.900	0.900	0.800	1.000																																															
**63**	0.600	0.600	0.700	0.700	0.700	0.800	0.500	0.700	0.500	0.700	0.600	0.400	0.500	0.400	0.500	0.600	0.500	0.400	0.400	0.500	0.500	0.700	0.235	0.600	0.400	0.400	0.700	0.400	0.400	0.600	0.700	0.700	0.800	0.600	0.800	0.700	0.800	0.600	0.700	0.700	0.500	0.800	0.600	0.444	0.700	0.700	0.700	0.600	0.800	1.000																																													
**64**	0.500	0.500	0.600	0.600	0.800	0.700	0.400	0.600	0.300	0.500	0.500	0.400	0.400	0.400	0.300	0.600	0.500	0.400	0.300	0.400	0.600	0.700	0.118	0.600	0.500	0.600	0.600	0.300	0.400	0.400	0.700	0.500	0.700	0.500	0.500	0.600	0.600	0.700	0.600	0.500	0.600	0.600	0.600	0.444	0.600	0.500	0.500	0.600	0.500	0.500	1.000																																												
**65**	0.600	0.600	0.700	0.600	0.700	0.800	0.500	0.900	0.500	0.700	0.600	0.300	0.500	0.300	0.500	0.600	0.700	0.600	0.400	0.500	0.600	0.800	0.235	0.600	0.400	0.500	0.700	0.400	0.600	0.600	0.700	0.500	0.600	0.500	0.600	0.800	0.800	0.700	0.500	0.700	0.600	0.600	0.500	0.556	0.700	0.700	0.700	0.800	0.700	0.700	0.600	1.000																																										
**68**	0.267	0.267	0.267	0.267	0.267	0.267	0.267	0.267	0.267	0.267	0.267	0.267	0.267	0.267	0.267	0.267	0.267	0.267	0.267	0.267	0.267	0.267	0.167	0.267	0.267	0.267	0.267	0.267	0.267	0.267	0.267	0.267	0.267	0.267	0.267	0.267	0.267	0.267	0.267	0.267	0.267	0.267	0.267	0.308	0.267	0.267	0.267	0.267	0.267	0.267	0.267	0.267	1.000																																									
**69**	0.500	0.500	0.500	0.600	0.500	0.500	0.700	0.500	0.700	0.400	0.500	0.600	0.600	0.500	0.700	0.500	0.700	0.700	0.800	0.600	0.300	0.600	0.353	0.600	0.600	0.500	0.500	0.800	0.700	0.600	0.500	0.700	0.500	0.600	0.600	0.600	0.500	0.500	0.600	0.400	0.400	0.400	0.500	0.556	0.600	0.600	0.700	0.700	0.700	0.500	0.400	0.500	0.267	1.000																																							
**72**	0.800	0.800	0.700	0.600	0.600	0.700	0.700	0.500	0.600	0.500	0.800	0.800	0.600	0.800	0.600	0.700	0.400	0.400	0.400	0.600	0.400	0.500	0.235	0.600	0.700	0.700	0.700	0.400	0.400	0.600	0.700	0.700	0.600	0.600	0.800	0.600	0.500	0.500	0.600	0.500	0.400	0.400	0.400	0.444	0.600	0.400	0.500	0.500	0.500	0.600	0.500	0.500	0.267	0.600	1.000																																						
**73**	0.600	0.600	0.600	0.600	0.500	0.600	0.400	0.600	0.400	0.800	0.600	0.300	0.400	0.300	0.400	0.500	0.400	0.400	0.300	0.400	0.400	0.500	0.118	0.500	0.400	0.400	0.600	0.300	0.400	0.500	0.500	0.500	0.500	0.600	0.600	0.500	0.700	0.500	0.400	0.800	0.400	0.600	0.500	0.444	0.600	0.500	0.600	0.400	0.500	0.600	0.500	0.600	0.267	0.400	0.500	1.000																																					
**74**	0.600	0.600	0.700	0.700	0.800	0.900	0.600	0.700	0.400	0.600	0.600	0.600	0.600	0.600	0.400	0.700	0.500	0.400	0.400	0.600	0.600	0.700	0.235	0.600	0.500	0.500	0.700	0.400	0.400	0.600	0.900	0.600	0.700	0.500	0.700	0.800	0.600	0.700	0.700	0.600	0.600	0.500	0.500	0.444	0.700	0.500	0.600	0.700	0.600	0.700	0.700	0.700	0.267	0.500	0.800	0.500	1.000																																				
**81**	0.700	0.700	0.800	0.800	0.800	0.900	0.500	0.700	0.500	0.700	0.700	0.500	0.500	0.500	0.500	0.700	0.500	0.400	0.400	0.500	0.600	0.700	0.235	0.700	0.500	0.500	0.800	0.400	0.400	0.600	0.800	0.800	0.900	0.600	0.900	0.700	0.800	0.700	0.800	0.700	0.600	0.700	0.500	0.444	0.700	0.700	0.700	0.600	0.800	0.900	0.600	0.700	0.267	0.500	0.700	0.600	0.800	1.000																																			
**84**	0.700	0.700	0.800	0.700	0.700	0.800	0.400	0.700	0.400	0.700	0.700	0.400	0.400	0.400	0.400	0.700	0.500	0.400	0.300	0.400	0.600	0.600	0.118	0.700	0.500	0.500	0.800	0.300	0.400	0.500	0.700	0.700	0.800	0.500	0.800	0.600	0.900	0.700	0.700	0.700	0.600	0.800	0.600	0.444	0.700	0.700	0.600	0.500	0.700	0.800	0.700	0.700	0.267	0.400	0.600	0.700	0.700	0.900	1.000																																		
**91**	0.778	0.778	0.778	0.667	0.667	0.778	0.556	0.667	0.444	0.667	0.778	0.556	0.556	0.556	0.444	0.778	0.444	0.444	0.444	0.556	0.444	0.556	0.267	0.556	0.667	0.667	0.778	0.444	0.444	0.778	0.889	0.667	0.667	0.556	0.778	0.778	0.556	0.667	0.778	0.667	0.444	0.444	0.444	0.625	0.667	0.556	0.556	0.556	0.556	0.667	0.556	0.556	0.308	0.556	0.778	0.556	0.778	0.778	0.667	1.000																																	
**92**	0.333	0.333	0.333	0.556	0.444	0.444	0.444	0.333	0.556	0.444	0.333	0.556	0.444	0.444	0.556	0.333	0.444	0.444	0.667	0.444	0.333	0.444	0.267	0.444	0.444	0.333	0.333	0.667	0.444	0.444	0.444	0.556	0.444	0.556	0.444	0.444	0.444	0.444	0.556	0.444	0.556	0.444	0.444	0.500	0.556	0.444	0.556	0.556	0.556	0.444	0.444	0.333	0.308	0.556	0.444	0.556	0.444	0.444	0.444	0.500	1.000																																
**97**	0.556	0.556	0.556	0.444	0.444	0.444	0.556	0.556	0.444	0.667	0.556	0.444	0.556	0.444	0.444	0.667	0.444	0.444	0.333	0.556	0.333	0.444	0.133	0.556	0.556	0.556	0.556	0.333	0.444	0.556	0.556	0.444	0.444	0.556	0.444	0.556	0.444	0.556	0.556	0.667	0.333	0.444	0.667	0.625	0.444	0.556	0.444	0.444	0.444	0.444	0.444	0.444	0.308	0.444	0.444	0.556	0.444	0.444	0.444	0.625	0.500	1.000																															
**98**	1.000	1.000	0.900	0.600	0.600	0.700	0.500	0.700	0.600	0.700	1.000	0.600	0.400	0.600	0.600	0.800	0.400	0.400	0.300	0.400	0.500	0.500	0.118	0.700	0.800	0.800	0.900	0.300	0.400	0.600	0.700	0.700	0.600	0.600	0.800	0.600	0.600	0.700	0.600	0.700	0.500	0.500	0.400	0.556	0.500	0.600	0.500	0.400	0.500	0.600	0.500	0.600	0.267	0.500	0.800	0.600	0.600	0.700	0.700	0.778	0.333	0.556	1.000																														
**99**	1.000	1.000	0.900	0.600	0.600	0.700	0.500	0.700	0.600	0.700	1.000	0.600	0.400	0.600	0.600	0.800	0.400	0.400	0.300	0.400	0.500	0.500	0.118	0.700	0.800	0.800	0.900	0.300	0.400	0.600	0.700	0.700	0.600	0.600	0.800	0.600	0.600	0.700	0.600	0.700	0.500	0.500	0.400	0.556	0.500	0.600	0.500	0.400	0.500	0.600	0.500	0.600	0.267	0.500	0.800	0.600	0.600	0.700	0.700	0.778	0.333	0.556	1.000	1.000																													
**100**	1.000	1.000	0.900	0.600	0.600	0.700	0.500	0.700	0.600	0.700	1.000	0.600	0.400	0.600	0.600	0.800	0.400	0.400	0.300	0.400	0.500	0.500	0.118	0.700	0.800	0.800	0.900	0.300	0.400	0.600	0.700	0.700	0.600	0.600	0.800	0.600	0.600	0.700	0.600	0.700	0.500	0.500	0.400	0.556	0.500	0.600	0.500	0.400	0.500	0.600	0.500	0.600	0.267	0.500	0.800	0.600	0.600	0.700	0.700	0.778	0.333	0.556	1.000	1.000	1.000																												
**101**	0.900	0.900	0.800	0.500	0.500	0.600	0.500	0.600	0.500	0.600	0.900	0.600	0.400	0.600	0.500	0.800	0.400	0.400	0.300	0.400	0.400	0.400	0.118	0.600	0.900	0.800	0.800	0.300	0.400	0.600	0.700	0.600	0.500	0.500	0.700	0.600	0.500	0.600	0.600	0.600	0.400	0.400	0.400	0.667	0.500	0.500	0.400	0.400	0.400	0.500	0.500	0.500	0.267	0.500	0.800	0.500	0.600	0.600	0.600	0.778	0.333	0.556	0.900	0.900	0.900	1.000																											
**102**	0.700	0.700	0.800	0.700	0.800	0.700	0.500	0.600	0.500	0.600	0.700	0.500	0.400	0.500	0.500	0.700	0.500	0.400	0.300	0.400	0.700	0.700	0.118	0.800	0.600	0.700	0.800	0.300	0.400	0.400	0.600	0.700	0.800	0.700	0.700	0.500	0.600	0.700	0.600	0.600	0.700	0.600	0.500	0.444	0.500	0.600	0.600	0.500	0.600	0.600	0.700	0.600	0.267	0.500	0.600	0.500	0.600	0.700	0.700	0.556	0.333	0.444	0.700	0.700	0.700	0.600	1.000																										
**103**	0.700	0.700	0.800	0.800	0.900	1.000	0.500	0.800	0.500	0.700	0.700	0.500	0.500	0.500	0.500	0.700	0.500	0.400	0.400	0.500	0.700	0.800	0.235	0.700	0.500	0.500	0.800	0.400	0.400	0.600	0.900	0.700	0.800	0.600	0.800	0.800	0.700	0.800	0.700	0.700	0.700	0.600	0.500	0.444	0.700	0.600	0.700	0.700	0.700	0.800	0.700	0.800	0.267	0.500	0.700	0.600	0.900	0.900	0.800	0.778	0.444	0.444	0.700	0.700	0.700	0.600	0.700	1.000																									
**104**	0.400	0.400	0.500	0.600	0.800	0.700	0.600	0.700	0.500	0.500	0.400	0.400	0.600	0.400	0.500	0.600	0.600	0.500	0.500	0.600	0.500	0.900	0.353	0.600	0.400	0.500	0.500	0.500	0.500	0.600	0.700	0.500	0.700	0.600	0.500	0.800	0.600	0.600	0.600	0.500	0.500	0.500	0.600	0.556	0.700	0.600	0.700	0.800	0.700	0.600	0.700	0.700	0.267	0.600	0.500	0.400	0.700	0.600	0.500	0.556	0.444	0.556	0.400	0.400	0.400	0.400	0.600	0.700	1.000																								
**105**	0.125	0.125	0.125	0.125	0.125	0.125	0.125	0.125	0.125	0.125	0.125	0.125	0.125	0.125	0.125	0.125	0.125	0.125	0.125	0.125	0.125	0.125	0.308	0.125	0.125	0.125	0.125	0.125	0.125	0.125	0.125	0.125	0.125	0.125	0.125	0.125	0.125	0.125	0.125	0.125	0.125	0.125	0.125	0.143	0.125	0.125	0.125	0.125	0.125	0.125	0.125	0.125	0.545	0.125	0.125	0.125	0.125	0.125	0.125	0.143	0.143	0.143	0.125	0.125	0.125	0.125	0.125	0.125	0.125	1.000																							
**134**	0.556	0.556	0.556	0.667	0.556	0.556	0.667	0.667	0.667	0.556	0.556	0.556	0.667	0.444	0.667	0.556	0.667	0.667	0.667	0.667	0.333	0.667	0.400	0.556	0.667	0.556	0.556	0.667	0.667	0.778	0.667	0.667	0.556	0.556	0.556	0.778	0.556	0.667	0.778	0.556	0.444	0.444	0.444	0.750	0.667	0.778	0.778	0.778	0.778	0.556	0.444	0.556	0.308	0.778	0.556	0.444	0.556	0.556	0.444	0.750	0.625	0.625	0.556	0.556	0.556	0.556	0.444	0.556	0.667	0.143	1.000																						
**146**	0.444	0.444	0.444	0.444	0.444	0.444	0.444	0.444	0.444	0.444	0.444	0.444	0.444	0.444	0.444	0.444	0.333	0.333	0.444	0.444	0.556	0.444	0.267	0.333	0.444	0.444	0.444	0.444	0.333	0.556	0.556	0.444	0.444	0.444	0.444	0.556	0.333	0.444	0.556	0.444	0.333	0.444	0.333	0.500	0.444	0.444	0.444	0.444	0.444	0.444	0.333	0.333	0.308	0.444	0.444	0.333	0.444	0.444	0.333	0.625	0.500	0.500	0.444	0.444	0.444	0.444	0.333	0.444	0.444	0.143	0.625	1.000																					
**152**	0.800	0.800	0.700	0.600	0.600	0.700	0.700	0.500	0.600	0.500	0.800	0.800	0.600	0.800	0.600	0.700	0.400	0.400	0.400	0.600	0.400	0.500	0.235	0.600	0.700	0.700	0.700	0.400	0.400	0.600	0.700	0.700	0.600	0.600	0.800	0.600	0.500	0.500	0.600	0.500	0.400	0.400	0.400	0.444	0.600	0.400	0.500	0.500	0.500	0.600	0.500	0.500	0.267	0.600	1.000	0.500	0.800	0.700	0.600	0.778	0.444	0.444	0.800	0.800	0.800	0.800	0.600	0.700	0.500	0.125	0.556	0.444	1.000																				
**154**	0.600	0.600	0.700	0.600	0.600	0.700	0.500	0.700	0.400	0.800	0.600	0.400	0.500	0.400	0.400	0.700	0.500	0.400	0.400	0.500	0.400	0.600	0.235	0.500	0.500	0.500	0.700	0.400	0.400	0.700	0.800	0.500	0.600	0.700	0.600	0.800	0.600	0.600	0.700	0.800	0.400	0.500	0.500	0.556	0.700	0.600	0.600	0.600	0.600	0.700	0.500	0.600	0.267	0.500	0.600	0.600	0.700	0.700	0.600	0.778	0.556	0.667	0.600	0.600	0.600	0.600	0.500	0.700	0.600	0.125	0.667	0.556	0.600	1.000																			
**155**	0.700	0.700	0.600	0.500	0.600	0.500	0.600	0.600	0.700	0.500	0.700	0.500	0.500	0.500	0.700	0.500	0.500	0.500	0.400	0.500	0.500	0.700	0.235	0.600	0.600	0.700	0.600	0.400	0.500	0.500	0.400	0.600	0.600	0.700	0.600	0.500	0.600	0.500	0.400	0.500	0.500	0.500	0.400	0.556	0.500	0.600	0.600	0.500	0.600	0.500	0.500	0.600	0.267	0.600	0.600	0.500	0.400	0.500	0.500	0.444	0.333	0.444	0.700	0.700	0.700	0.600	0.800	0.500	0.600	0.125	0.556	0.333	0.600	0.400	1.000																		
**156**	0.600	0.600	0.700	0.700	0.700	0.700	0.400	0.700	0.400	0.600	0.600	0.500	0.400	0.400	0.400	0.800	0.600	0.500	0.400	0.400	0.600	0.600	0.118	0.800	0.700	0.600	0.700	0.400	0.500	0.500	0.800	0.600	0.600	0.400	0.500	0.700	0.500	0.900	0.800	0.600	0.700	0.500	0.600	0.556	0.500	0.700	0.600	0.700	0.600	0.500	0.700	0.600	0.267	0.500	0.500	0.400	0.700	0.600	0.600	0.667	0.444	0.667	0.600	0.600	0.600	0.600	0.600	0.700	0.700	0.125	0.667	0.444	0.500	0.600	0.400	1.000																	
**157**	0.700	0.700	0.800	0.700	0.800	0.900	0.500	0.800	0.400	0.700	0.700	0.500	0.500	0.500	0.400	0.800	0.500	0.400	0.400	0.500	0.600	0.700	0.235	0.600	0.600	0.600	0.800	0.400	0.400	0.700	1.000	0.600	0.700	0.500	0.700	0.900	0.600	0.800	0.800	0.700	0.600	0.500	0.500	0.556	0.700	0.600	0.600	0.700	0.600	0.700	0.700	0.700	0.267	0.500	0.700	0.500	0.900	0.800	0.700	0.889	0.444	0.556	0.700	0.700	0.700	0.700	0.600	0.900	0.700	0.125	0.667	0.556	0.700	0.800	0.400	0.800	1.000																
**158**	0.300	0.300	0.300	0.500	0.400	0.400	0.500	0.400	0.600	0.300	0.300	0.500	0.500	0.400	0.600	0.300	0.600	0.700	1.000	0.500	0.300	0.500	0.353	0.400	0.400	0.300	0.300	1.000	0.700	0.500	0.400	0.500	0.400	0.400	0.400	0.500	0.400	0.400	0.500	0.300	0.500	0.300	0.400	0.556	0.500	0.500	0.600	0.600	0.600	0.400	0.300	0.400	0.267	0.800	0.400	0.300	0.400	0.400	0.300	0.444	0.667	0.333	0.300	0.300	0.300	0.300	0.300	0.400	0.500	0.125	0.667	0.444	0.400	0.400	0.400	0.400	0.400	1.000														
**159**	0.400	0.400	0.500	0.400	0.500	0.500	0.400	0.700	0.400	0.500	0.400	0.300	0.400	0.300	0.400	0.500	0.500	0.400	0.600	0.400	0.500	0.600	0.235	0.400	0.400	0.400	0.500	0.600	0.400	0.500	0.600	0.300	0.400	0.300	0.300	0.700	0.500	0.600	0.500	0.500	0.600	0.400	0.400	0.667	0.500	0.600	0.500	0.600	0.500	0.400	0.500	0.600	0.267	0.500	0.300	0.300	0.500	0.400	0.400	0.444	0.444	0.444	0.400	0.400	0.400	0.400	0.400	0.500	0.600	0.125	0.556	0.444	0.300	0.500	0.400	0.600	0.600	0.600	1.000													
**160**	1.000	1.000	0.900	0.600	0.600	0.700	0.500	0.700	0.600	0.700	1.000	0.600	0.400	0.600	0.600	0.800	0.400	0.400	0.300	0.400	0.500	0.500	0.118	0.700	0.800	0.800	0.900	0.300	0.400	0.600	0.700	0.700	0.600	0.600	0.800	0.600	0.600	0.700	0.600	0.700	0.500	0.500	0.400	0.556	0.500	0.600	0.500	0.400	0.500	0.600	0.500	0.600	0.267	0.500	0.800	0.600	0.600	0.700	0.700	0.778	0.333	0.556	1.000	1.000	1.000	0.900	0.700	0.700	0.400	0.125	0.556	0.444	0.800	0.600	0.700	0.600	0.700	0.300	0.400	1.000												
**161**	1.000	1.000	0.900	0.600	0.600	0.700	0.500	0.700	0.600	0.700	1.000	0.600	0.400	0.600	0.600	0.800	0.400	0.400	0.300	0.400	0.500	0.500	0.118	0.700	0.800	0.800	0.900	0.300	0.400	0.600	0.700	0.700	0.600	0.600	0.800	0.600	0.600	0.700	0.600	0.700	0.500	0.500	0.400	0.556	0.500	0.600	0.500	0.400	0.500	0.600	0.500	0.600	0.267	0.500	0.800	0.600	0.600	0.700	0.700	0.778	0.333	0.556	1.000	1.000	1.000	0.900	0.700	0.700	0.400	0.125	0.556	0.444	0.800	0.600	0.700	0.600	0.700	0.300	0.400	1.000	1.000											
**162**	0.800	0.800	0.900	0.700	0.700	0.800	0.500	0.700	0.500	0.700	0.800	0.500	0.400	0.500	0.500	0.900	0.500	0.400	0.300	0.400	0.600	0.600	0.118	0.900	0.600	0.600	0.900	0.300	0.400	0.500	0.700	0.700	0.700	0.600	0.800	0.600	0.700	0.700	0.600	0.700	0.600	0.600	0.600	0.444	0.600	0.600	0.600	0.500	0.600	0.700	0.600	0.700	0.267	0.500	0.700	0.600	0.700	0.800	0.800	0.667	0.333	0.556	0.800	0.800	0.800	0.700	0.800	0.800	0.600	0.125	0.444	0.333	0.700	0.600	0.600	0.700	0.700	0.300	0.400	0.800	0.800	1.000									
**163**	0.600	0.600	0.700	0.600	0.700	0.800	0.500	0.900	0.500	0.700	0.600	0.300	0.500	0.300	0.500	0.600	0.600	0.500	0.400	0.500	0.700	0.800	0.235	0.600	0.400	0.400	0.700	0.400	0.500	0.600	0.700	0.500	0.600	0.500	0.600	0.800	0.800	0.700	0.500	0.700	0.700	0.600	0.500	0.556	0.700	0.700	0.700	0.700	0.700	0.700	0.600	0.900	0.267	0.500	0.500	0.600	0.700	0.700	0.700	0.556	0.333	0.444	0.600	0.600	0.600	0.500	0.700	0.800	0.700	0.125	0.556	0.333	0.500	0.600	0.700	0.600	0.700	0.400	0.600	0.600	0.600	0.700	1.000								
**164**	0.400	0.400	0.400	0.500	0.400	0.400	0.500	0.500	0.500	0.400	0.400	0.400	0.500	0.300	0.500	0.400	0.900	1.000	0.700	0.500	0.300	0.500	0.235	0.500	0.500	0.500	0.400	0.700	1.000	0.500	0.400	0.500	0.400	0.400	0.400	0.500	0.500	0.500	0.500	0.400	0.400	0.400	0.500	0.556	0.500	0.600	0.600	0.700	0.600	0.400	0.400	0.600	0.267	0.700	0.400	0.400	0.400	0.400	0.400	0.444	0.444	0.444	0.400	0.400	0.400	0.400	0.400	0.400	0.500	0.125	0.667	0.333	0.400	0.400	0.500	0.500	0.400	0.700	0.400	0.400	0.400	0.400	0.500	1.000							
**165**	0.600	0.600	0.500	0.500	0.500	0.500	0.700	0.300	0.700	0.300	0.600	0.900	0.600	1.000	0.700	0.500	0.300	0.300	0.400	0.600	0.400	0.400	0.235	0.500	0.600	0.600	0.500	0.400	0.300	0.400	0.500	0.600	0.500	0.500	0.600	0.400	0.300	0.400	0.500	0.300	0.400	0.300	0.400	0.333	0.400	0.300	0.400	0.400	0.400	0.400	0.400	0.300	0.267	0.500	0.800	0.300	0.600	0.500	0.400	0.556	0.444	0.444	0.600	0.600	0.600	0.600	0.500	0.500	0.400	0.125	0.444	0.444	0.800	0.400	0.500	0.400	0.500	0.400	0.300	0.600	0.600	0.500	0.300	0.300	1.000						
**166**	0.700	0.700	0.800	0.700	0.600	0.700	0.400	0.800	0.400	1.000	0.700	0.300	0.400	0.300	0.400	0.700	0.500	0.400	0.300	0.400	0.500	0.600	0.118	0.600	0.500	0.500	0.800	0.300	0.400	0.600	0.700	0.500	0.600	0.600	0.600	0.700	0.700	0.700	0.600	1.000	0.500	0.600	0.500	0.556	0.600	0.700	0.500	0.600	0.500	0.700	0.500	0.700	0.267	0.400	0.500	0.800	0.600	0.700	0.700	0.667	0.444	0.667	0.700	0.700	0.700	0.600	0.600	0.700	0.500	0.125	0.556	0.444	0.500	0.800	0.500	0.600	0.700	0.300	0.500	0.700	0.700	0.700	0.700	0.400	0.300	1.000					
**167**	0.800	0.800	0.700	0.400	0.400	0.500	0.500	0.600	0.500	0.700	0.800	0.500	0.400	0.500	0.500	0.700	0.400	0.400	0.300	0.400	0.300	0.400	0.118	0.500	0.800	0.700	0.700	0.300	0.400	0.600	0.600	0.500	0.400	0.600	0.600	0.600	0.500	0.500	0.500	0.700	0.300	0.400	0.400	0.667	0.500	0.500	0.400	0.400	0.400	0.500	0.400	0.500	0.267	0.500	0.700	0.600	0.500	0.600	0.500	0.667	0.444	0.667	0.800	0.800	0.800	0.900	0.500	0.500	0.400	0.125	0.556	0.444	0.700	0.700	0.600	0.500	0.600	0.300	0.400	0.800	0.800	0.600	0.500	0.400	0.500	0.700	1.000				
**168**	0.600	0.600	0.700	0.900	0.900	0.900	0.500	0.700	0.600	0.600	0.600	0.600	0.500	0.500	0.600	0.600	0.600	0.500	0.500	0.500	0.700	0.800	0.235	0.800	0.600	0.500	0.700	0.500	0.500	0.500	0.800	0.800	0.800	0.600	0.700	0.700	0.600	0.900	0.800	0.600	0.800	0.600	0.500	0.444	0.600	0.700	0.800	0.600	0.500	0.700	0.700	0.700	0.267	0.600	0.600	0.500	0.800	0.800	0.700	0.667	0.556	0.444	0.600	0.600	0.600	0.500	0.700	0.900	0.700	0.125	0.667	0.444	0.600	0.600	0.500	0.800	0.800	0.500	0.500	0.600	0.600	0.700	0.700	0.500	0.500	0.600	0.400	1.000			
**169**	0.900	0.900	0.800	0.500	0.500	0.600	0.500	0.600	0.500	0.600	0.900	0.600	0.400	0.600	0.500	0.800	0.400	0.400	0.300	0.400	0.400	0.400	0.118	0.600	0.900	0.800	0.800	0.300	0.400	0.600	0.700	0.600	0.500	0.500	0.700	0.600	0.500	0.600	0.600	0.600	0.400	0.400	0.400	0.667	0.500	0.500	0.400	0.400	0.400	0.500	0.500	0.500	0.267	0.500	0.800	0.500	0.600	0.600	0.600	0.778	0.333	0.556	0.900	0.900	0.900	1.000	0.600	0.600	0.400	0.125	0.556	0.444	0.800	0.600	0.600	0.600	0.700	0.300	0.400	0.900	0.900	0.700	0.500	0.400	0.600	0.600	0.900	0.500	1.000	
**170**	0.400	0.400	0.500	0.600	0.800	0.700	0.500	0.600	0.400	0.600	0.400	0.400	0.500	0.400	0.400	0.600	0.500	0.400	0.400	0.500	0.500	0.800	0.235	0.600	0.400	0.500	0.500	0.400	0.400	0.500	0.700	0.500	0.700	0.700	0.500	0.700	0.500	0.600	0.600	0.600	0.500	0.500	0.600	0.444	0.600	0.500	0.600	0.700	0.600	0.600	0.700	0.600	0.267	0.500	0.500	0.500	0.700	0.600	0.500	0.556	0.556	0.667	0.400	0.400	0.400	0.400	0.600	0.700	0.900	0.125	0.556	0.444	0.500	0.700	0.500	0.700	0.700	0.400	0.500	0.400	0.400	0.600	0.600	0.400	0.400	0.600	0.500	0.700	0.400	1.000
