# Supplementary material for: Assessment of Wild Rocket (Diplotaxis tenuifolia (L.) DC.) Germplasm Accessions by NGS Identified SSR and SNP Markers
Source: Plants (Basel). 2022 Dec 12;11(24):3482. doi: 10.3390/plants11243482 (PMC9783343; doi:10.3390/plants11243482)
Supplement: Supplementary file 1 [file Table S5.pdf › Table S5.pdf]

Table SM5. Genetic similarity matrix (SNP-CAPS markers)

Accessions:	1	3	4	5	6	7	8	9	10	12	15	16	17	18	19	20	21	22	23	24	26	27	29	32	35	36	37	38	39	40	41	42	43	44	45	46	47	48	49	51	52	54	55	56	57	58	59	60	61	63	64	65	68	69	72	73	74	81	84	91	92	97	98	99	100	101	102	103	104	105	134	146	152	154	155	156	157	158	159	160	161	162	163	164	165	166	167	168	169	170
**1**	1.000
**3**	0.889	1.000
**4**	1.000	0.889	1.000
**5**	0.944	0.944	0.944	1.000
**6**	0.778	0.778	0.778	0.722	1.000
**7**	0.833	0.833	0.833	0.778	0.833	1.000
**8**	0.833	0.722	0.833	0.778	0.833	0.889	1.000
**9**	0.889	0.778	0.889	0.833	0.889	0.722	0.833	1.000
**10**	0.889	0.778	0.889	0.833	0.889	0.833	0.944	0.889	1.000
**12**	0.944	0.944	0.944	0.889	0.833	0.889	0.778	0.833	0.833	1.000
**15**	1.000	0.889	1.000	0.944	0.778	0.833	0.833	0.889	0.889	0.944	1.000
**16**	0.722	0.722	0.722	0.667	0.833	0.778	0.778	0.722	0.833	0.778	0.722	1.000
**17**	0.889	0.778	0.889	0.833	0.889	0.833	0.944	0.889	1.000	0.833	0.889	0.833	1.000
**18**	0.722	0.611	0.722	0.667	0.833	0.667	0.778	0.833	0.833	0.667	0.722	0.889	0.833	1.000
**19**	0.889	0.778	0.889	0.833	0.889	0.833	0.944	0.889	1.000	0.833	0.889	0.833	1.000	0.833	1.000
**20**	0.778	0.889	0.778	0.833	0.778	0.722	0.611	0.778	0.667	0.833	0.778	0.722	0.667	0.722	0.667	1.000
**21**	0.833	0.833	0.833	0.889	0.722	0.667	0.667	0.833	0.722	0.778	0.833	0.667	0.722	0.778	0.722	0.944	1.000
**22**	0.778	0.778	0.778	0.833	0.667	0.722	0.722	0.778	0.667	0.722	0.778	0.611	0.667	0.722	0.667	0.889	0.944	1.000
**23**	0.889	0.778	0.889	0.833	0.778	0.722	0.722	0.889	0.778	0.833	0.889	0.722	0.778	0.833	0.778	0.889	0.944	0.889	1.000
**24**	0.889	0.778	0.889	0.833	0.889	0.833	0.944	0.889	1.000	0.833	0.889	0.833	1.000	0.833	1.000	0.667	0.722	0.667	0.778	1.000
**26**	0.833	0.944	0.833	0.889	0.722	0.778	0.667	0.722	0.722	0.889	0.833	0.667	0.722	0.556	0.722	0.833	0.778	0.722	0.722	0.722	1.000
**27**	0.889	0.889	0.889	0.833	0.889	0.833	0.833	0.889	0.889	0.944	0.889	0.833	0.889	0.722	0.889	0.778	0.722	0.667	0.778	0.889	0.833	1.000
**29**	0.364	0.364	0.364	0.364	0.364	0.273	0.273	0.364	0.364	0.364	0.364	0.364	0.364	0.364	0.364	0.364	0.364	0.273	0.364	0.364	0.364	0.364	1.000
**32**	0.889	0.889	0.889	0.944	0.778	0.833	0.833	0.778	0.889	0.833	0.889	0.722	0.889	0.722	0.889	0.778	0.833	0.778	0.778	0.889	0.833	0.778	0.364	1.000
**35**	0.833	0.944	0.833	0.889	0.833	0.778	0.667	0.833	0.722	0.889	0.833	0.667	0.722	0.667	0.722	0.944	0.889	0.833	0.833	0.722	0.889	0.833	0.364	0.833	1.000
**36**	0.833	0.833	0.833	0.778	0.944	0.778	0.778	0.944	0.833	0.889	0.833	0.778	0.833	0.778	0.833	0.833	0.778	0.722	0.833	0.833	0.778	0.944	0.364	0.722	0.889	1.000
**37**	0.889	1.000	0.889	0.944	0.778	0.833	0.722	0.778	0.778	0.944	0.889	0.722	0.778	0.611	0.778	0.889	0.833	0.778	0.778	0.778	0.944	0.889	0.364	0.889	0.944	0.833	1.000
**38**	0.889	0.778	0.889	0.833	0.778	0.722	0.722	0.889	0.778	0.833	0.889	0.722	0.778	0.833	0.778	0.889	0.944	0.889	1.000	0.778	0.722	0.778	0.364	0.778	0.833	0.833	0.778	1.000
**39**	0.833	0.833	0.833	0.889	0.722	0.667	0.667	0.833	0.722	0.778	0.833	0.667	0.722	0.778	0.722	0.944	1.000	0.944	0.944	0.722	0.778	0.722	0.364	0.833	0.889	0.778	0.833	0.944	1.000
**40**	0.833	0.722	0.833	0.778	0.833	0.889	1.000	0.833	0.944	0.778	0.833	0.778	0.944	0.778	0.944	0.611	0.667	0.722	0.722	0.944	0.667	0.833	0.273	0.833	0.667	0.778	0.722	0.722	0.667	1.000
**41**	0.889	0.889	0.889	0.833	0.889	0.833	0.833	0.889	0.889	0.944	0.889	0.833	0.889	0.722	0.889	0.778	0.722	0.667	0.778	0.889	0.833	1.000	0.364	0.778	0.833	0.944	0.889	0.778	0.722	0.833	1.000
**42**	0.944	0.833	0.944	0.889	0.833	0.889	0.889	0.833	0.944	0.889	0.944	0.778	0.944	0.778	0.944	0.722	0.778	0.722	0.833	0.944	0.778	0.833	0.364	0.944	0.778	0.778	0.833	0.833	0.778	0.889	0.833	1.000
**43**	0.889	0.778	0.889	0.833	0.889	0.833	0.944	0.889	1.000	0.833	0.889	0.833	1.000	0.833	1.000	0.667	0.722	0.667	0.778	1.000	0.722	0.889	0.364	0.889	0.722	0.833	0.778	0.778	0.722	0.944	0.889	0.944	1.000
**44**	0.778	0.778	0.778	0.722	1.000	0.833	0.833	0.889	0.889	0.833	0.778	0.833	0.889	0.833	0.889	0.778	0.722	0.667	0.778	0.889	0.722	0.889	0.364	0.778	0.833	0.944	0.778	0.778	0.722	0.833	0.889	0.833	0.889	1.000
**45**	0.889	0.889	0.889	0.944	0.778	0.722	0.722	0.889	0.778	0.833	0.889	0.611	0.778	0.722	0.778	0.889	0.944	0.889	0.889	0.778	0.833	0.778	0.364	0.889	0.944	0.833	0.889	0.889	0.944	0.722	0.778	0.833	0.778	0.778	1.000
**46**	0.889	0.889	0.889	0.944	0.778	0.722	0.833	0.889	0.889	0.833	0.889	0.722	0.889	0.722	0.889	0.778	0.833	0.778	0.778	0.889	0.833	0.889	0.364	0.889	0.833	0.833	0.889	0.778	0.833	0.833	0.889	0.833	0.889	0.778	0.889	1.000
**47**	0.889	0.889	0.889	0.944	0.778	0.722	0.833	0.889	0.889	0.833	0.889	0.722	0.889	0.722	0.889	0.778	0.833	0.778	0.778	0.889	0.833	0.889	0.364	0.889	0.833	0.833	0.889	0.778	0.833	0.833	0.889	0.833	0.889	0.778	0.889	1.000	1.000
**48**	0.778	0.889	0.778	0.833	0.889	0.833	0.833	0.778	0.889	0.833	0.778	0.833	0.889	0.722	0.889	0.778	0.722	0.667	0.667	0.889	0.833	0.889	0.364	0.889	0.833	0.833	0.889	0.667	0.722	0.833	0.889	0.833	0.889	0.889	0.778	0.889	0.889	1.000
**49**	0.833	0.833	0.833	0.889	0.833	0.667	0.778	0.944	0.833	0.778	0.833	0.667	0.833	0.778	0.833	0.833	0.889	0.833	0.833	0.833	0.778	0.833	0.364	0.833	0.889	0.889	0.833	0.833	0.889	0.778	0.833	0.778	0.833	0.833	0.944	0.944	0.944	0.833	1.000
**51**	0.889	0.889	0.889	0.833	0.889	0.833	0.833	0.889	0.889	0.944	0.889	0.833	0.889	0.722	0.889	0.778	0.722	0.667	0.778	0.889	0.833	1.000	0.364	0.778	0.833	0.944	0.889	0.778	0.722	0.833	1.000	0.833	0.889	0.889	0.778	0.889	0.889	0.889	0.833	1.000
**52**	0.778	0.778	0.778	0.722	1.000	0.833	0.833	0.889	0.889	0.833	0.778	0.833	0.889	0.833	0.889	0.778	0.722	0.667	0.778	0.889	0.722	0.889	0.364	0.778	0.833	0.944	0.778	0.778	0.722	0.833	0.889	0.833	0.889	1.000	0.778	0.778	0.778	0.889	0.833	0.889	1.000
**54**	0.889	0.889	0.889	0.833	0.889	0.833	0.833	0.889	0.889	0.944	0.889	0.833	0.889	0.722	0.889	0.778	0.722	0.667	0.778	0.889	0.833	1.000	0.364	0.778	0.833	0.944	0.889	0.778	0.722	0.833	1.000	0.833	0.889	0.889	0.778	0.889	0.889	0.889	0.833	1.000	0.889	1.000
**55**	0.944	0.833	0.944	0.889	0.722	0.778	0.778	0.833	0.833	0.889	0.944	0.667	0.833	0.667	0.833	0.722	0.778	0.722	0.833	0.833	0.778	0.833	0.364	0.833	0.778	0.778	0.833	0.833	0.778	0.778	0.833	0.889	0.833	0.722	0.833	0.833	0.833	0.722	0.778	0.833	0.722	0.833	1.000
**56**	0.833	0.833	0.833	0.889	0.611	0.667	0.667	0.722	0.722	0.778	0.833	0.556	0.722	0.556	0.722	0.722	0.778	0.722	0.722	0.722	0.889	0.722	0.364	0.833	0.778	0.667	0.833	0.722	0.778	0.667	0.722	0.778	0.722	0.611	0.833	0.833	0.833	0.722	0.778	0.722	0.611	0.722	0.889	1.000
**57**	0.944	0.944	0.944	0.889	0.833	0.889	0.778	0.833	0.833	1.000	0.944	0.778	0.833	0.667	0.833	0.833	0.778	0.722	0.833	0.833	0.889	0.944	0.364	0.833	0.889	0.889	0.944	0.833	0.778	0.778	0.944	0.889	0.833	0.833	0.833	0.833	0.833	0.833	0.778	0.944	0.833	0.944	0.889	0.778	1.000
**58**	0.889	1.000	0.889	0.944	0.778	0.833	0.722	0.778	0.778	0.944	0.889	0.722	0.778	0.611	0.778	0.889	0.833	0.778	0.778	0.778	0.944	0.889	0.364	0.889	0.944	0.833	1.000	0.778	0.833	0.722	0.889	0.833	0.778	0.778	0.889	0.889	0.889	0.889	0.833	0.889	0.778	0.889	0.833	0.833	0.944	1.000
**59**	1.000	0.889	1.000	0.944	0.778	0.833	0.833	0.889	0.889	0.944	1.000	0.722	0.889	0.722	0.889	0.778	0.833	0.778	0.889	0.889	0.833	0.889	0.364	0.889	0.833	0.833	0.889	0.889	0.833	0.833	0.889	0.944	0.889	0.778	0.889	0.889	0.889	0.778	0.833	0.889	0.778	0.889	0.944	0.833	0.944	0.889	1.000
**60**	0.889	1.000	0.889	0.944	0.778	0.833	0.722	0.778	0.778	0.944	0.889	0.722	0.778	0.611	0.778	0.889	0.833	0.778	0.778	0.778	0.944	0.889	0.364	0.889	0.944	0.833	1.000	0.778	0.833	0.722	0.889	0.833	0.778	0.778	0.889	0.889	0.889	0.889	0.833	0.889	0.778	0.889	0.833	0.833	0.944	1.000	0.889	1.000
**61**	0.944	0.833	0.944	0.889	0.833	0.778	0.778	0.944	0.833	0.889	0.944	0.667	0.833	0.778	0.833	0.833	0.889	0.833	0.944	0.833	0.778	0.833	0.364	0.833	0.889	0.889	0.833	0.944	0.889	0.778	0.833	0.889	0.833	0.833	0.944	0.833	0.833	0.722	0.889	0.833	0.833	0.833	0.889	0.778	0.889	0.833	0.944	0.833	1.000
**63**	1.000	0.889	1.000	0.944	0.778	0.833	0.833	0.889	0.889	0.944	1.000	0.722	0.889	0.722	0.889	0.778	0.833	0.778	0.889	0.889	0.833	0.889	0.364	0.889	0.833	0.833	0.889	0.889	0.833	0.833	0.889	0.944	0.889	0.778	0.889	0.889	0.889	0.778	0.833	0.889	0.778	0.889	0.944	0.833	0.944	0.889	1.000	0.889	0.944	1.000
**64**	1.000	0.889	1.000	0.944	0.778	0.833	0.833	0.889	0.889	0.944	1.000	0.722	0.889	0.722	0.889	0.778	0.833	0.778	0.889	0.889	0.833	0.889	0.364	0.889	0.833	0.833	0.889	0.889	0.833	0.833	0.889	0.944	0.889	0.778	0.889	0.889	0.889	0.778	0.833	0.889	0.778	0.889	0.944	0.833	0.944	0.889	1.000	0.889	0.944	1.000	1.000
**65**	1.000	0.889	1.000	0.944	0.778	0.833	0.833	0.889	0.889	0.944	1.000	0.722	0.889	0.722	0.889	0.778	0.833	0.778	0.889	0.889	0.833	0.889	0.364	0.889	0.833	0.833	0.889	0.889	0.833	0.833	0.889	0.944	0.889	0.778	0.889	0.889	0.889	0.778	0.833	0.889	0.778	0.889	0.944	0.833	0.944	0.889	1.000	0.889	0.944	1.000	1.000	1.000
**68**	0.364	0.364	0.364	0.364	0.364	0.273	0.273	0.364	0.364	0.364	0.364	0.364	0.364	0.364	0.364	0.364	0.364	0.273	0.364	0.364	0.364	0.364	1.000	0.364	0.364	0.364	0.364	0.364	0.364	0.273	0.364	0.364	0.364	0.364	0.364	0.364	0.364	0.364	0.364	0.364	0.364	0.364	0.364	0.364	0.364	0.364	0.364	0.364	0.364	0.364	0.364	0.364	1.000
**69**	0.778	0.889	0.778	0.833	0.889	0.833	0.833	0.778	0.889	0.833	0.778	0.833	0.889	0.722	0.889	0.778	0.722	0.667	0.667	0.889	0.833	0.889	0.364	0.889	0.833	0.833	0.889	0.667	0.722	0.833	0.889	0.833	0.889	0.889	0.778	0.889	0.889	1.000	0.833	0.889	0.889	0.889	0.722	0.722	0.833	0.889	0.778	0.889	0.722	0.778	0.778	0.778	0.364	1.000
**72**	0.833	0.833	0.833	0.889	0.833	0.778	0.889	0.833	0.944	0.778	0.833	0.778	0.944	0.778	0.944	0.722	0.778	0.722	0.722	0.944	0.778	0.833	0.364	0.944	0.778	0.778	0.833	0.722	0.778	0.889	0.833	0.889	0.944	0.833	0.833	0.944	0.944	0.944	0.889	0.833	0.833	0.833	0.778	0.778	0.778	0.833	0.833	0.833	0.778	0.833	0.833	0.833	0.364	0.944	1.000
**73**	0.889	1.000	0.889	0.944	0.778	0.833	0.722	0.778	0.778	0.944	0.889	0.722	0.778	0.611	0.778	0.889	0.833	0.778	0.778	0.778	0.944	0.889	0.364	0.889	0.944	0.833	1.000	0.778	0.833	0.722	0.889	0.833	0.778	0.778	0.889	0.889	0.889	0.889	0.833	0.889	0.778	0.889	0.833	0.833	0.944	1.000	0.889	1.000	0.833	0.889	0.889	0.889	0.364	0.889	0.833	1.000
**74**	0.889	0.778	0.889	0.833	0.889	0.833	0.833	0.889	0.889	0.833	0.889	0.722	0.889	0.833	0.889	0.778	0.833	0.778	0.889	0.889	0.722	0.778	0.364	0.889	0.833	0.833	0.778	0.889	0.833	0.833	0.778	0.944	0.889	0.889	0.889	0.778	0.778	0.778	0.833	0.778	0.889	0.778	0.833	0.722	0.833	0.778	0.889	0.778	0.944	0.889	0.889	0.889	0.364	0.778	0.833	0.778	1.000
**81**	0.944	0.833	0.944	0.889	0.833	0.778	0.889	0.944	0.944	0.889	0.944	0.778	0.944	0.778	0.944	0.722	0.778	0.722	0.833	0.944	0.778	0.944	0.364	0.833	0.778	0.889	0.833	0.833	0.778	0.889	0.944	0.889	0.944	0.833	0.833	0.944	0.944	0.833	0.889	0.944	0.833	0.944	0.889	0.778	0.889	0.833	0.944	0.833	0.889	0.944	0.944	0.944	0.364	0.833	0.889	0.833	0.833	1.000
**84**	0.944	0.944	0.944	1.000	0.722	0.778	0.778	0.833	0.833	0.889	0.944	0.667	0.833	0.667	0.833	0.833	0.889	0.833	0.833	0.833	0.889	0.833	0.364	0.944	0.889	0.778	0.944	0.833	0.889	0.778	0.833	0.889	0.833	0.722	0.944	0.944	0.944	0.833	0.889	0.833	0.722	0.833	0.889	0.889	0.889	0.944	0.944	0.944	0.889	0.944	0.944	0.944	0.364	0.833	0.889	0.944	0.833	0.889	1.000
**91**	0.889	0.778	0.889	0.833	0.778	0.722	0.833	0.889	0.889	0.833	0.889	0.722	0.889	0.722	0.889	0.667	0.722	0.667	0.778	0.889	0.722	0.889	0.364	0.778	0.722	0.833	0.778	0.778	0.722	0.833	0.889	0.833	0.889	0.778	0.778	0.889	0.889	0.778	0.833	0.889	0.778	0.889	0.944	0.833	0.833	0.778	0.889	0.778	0.833	0.889	0.889	0.889	0.364	0.778	0.833	0.778	0.778	0.944	0.833	1.000
**92**	1.000	0.889	1.000	0.944	0.778	0.833	0.833	0.889	0.889	0.944	1.000	0.722	0.889	0.722	0.889	0.778	0.833	0.778	0.889	0.889	0.833	0.889	0.364	0.889	0.833	0.833	0.889	0.889	0.833	0.833	0.889	0.944	0.889	0.778	0.889	0.889	0.889	0.778	0.833	0.889	0.778	0.889	0.944	0.833	0.944	0.889	1.000	0.889	0.944	1.000	1.000	1.000	0.364	0.778	0.833	0.889	0.889	0.944	0.944	0.889	1.000
**97**	0.889	0.889	0.889	0.833	0.889	0.833	0.722	0.889	0.778	0.944	0.889	0.722	0.778	0.722	0.778	0.889	0.833	0.778	0.889	0.778	0.833	0.889	0.364	0.778	0.944	0.944	0.889	0.889	0.833	0.722	0.889	0.833	0.778	0.889	0.889	0.778	0.778	0.778	0.833	0.889	0.889	0.889	0.833	0.722	0.944	0.889	0.889	0.889	0.944	0.889	0.889	0.889	0.364	0.778	0.722	0.889	0.889	0.833	0.833	0.778	0.889	1.000
**98**	1.000	0.889	1.000	0.944	0.778	0.833	0.833	0.889	0.889	0.944	1.000	0.722	0.889	0.722	0.889	0.778	0.833	0.778	0.889	0.889	0.833	0.889	0.364	0.889	0.833	0.833	0.889	0.889	0.833	0.833	0.889	0.944	0.889	0.778	0.889	0.889	0.889	0.778	0.833	0.889	0.778	0.889	0.944	0.833	0.944	0.889	1.000	0.889	0.944	1.000	1.000	1.000	0.364	0.778	0.833	0.889	0.889	0.944	0.944	0.889	1.000	0.889	1.000
**99**	0.889	1.000	0.889	0.944	0.778	0.833	0.722	0.778	0.778	0.944	0.889	0.722	0.778	0.611	0.778	0.889	0.833	0.778	0.778	0.778	0.944	0.889	0.364	0.889	0.944	0.833	1.000	0.778	0.833	0.722	0.889	0.833	0.778	0.778	0.889	0.889	0.889	0.889	0.833	0.889	0.778	0.889	0.833	0.833	0.944	1.000	0.889	1.000	0.833	0.889	0.889	0.889	0.364	0.889	0.833	1.000	0.778	0.833	0.944	0.778	0.889	0.889	0.889	1.000
**100**	1.000	0.889	1.000	0.944	0.778	0.833	0.833	0.889	0.889	0.944	1.000	0.722	0.889	0.722	0.889	0.778	0.833	0.778	0.889	0.889	0.833	0.889	0.364	0.889	0.833	0.833	0.889	0.889	0.833	0.833	0.889	0.944	0.889	0.778	0.889	0.889	0.889	0.778	0.833	0.889	0.778	0.889	0.944	0.833	0.944	0.889	1.000	0.889	0.944	1.000	1.000	1.000	0.364	0.778	0.833	0.889	0.889	0.944	0.944	0.889	1.000	0.889	1.000	0.889	1.000
**101**	0.833	0.944	0.833	0.889	0.833	0.778	0.667	0.833	0.722	0.889	0.833	0.667	0.722	0.667	0.722	0.944	0.889	0.833	0.833	0.722	0.889	0.833	0.364	0.833	1.000	0.889	0.944	0.833	0.889	0.667	0.833	0.778	0.722	0.833	0.944	0.833	0.833	0.833	0.889	0.833	0.833	0.833	0.778	0.778	0.889	0.944	0.833	0.944	0.889	0.833	0.833	0.833	0.364	0.833	0.778	0.944	0.833	0.778	0.889	0.722	0.833	0.944	0.833	0.944	0.833	1.000
**102**	0.889	1.000	0.889	0.944	0.778	0.833	0.722	0.778	0.778	0.944	0.889	0.722	0.778	0.611	0.778	0.889	0.833	0.778	0.778	0.778	0.944	0.889	0.364	0.889	0.944	0.833	1.000	0.778	0.833	0.722	0.889	0.833	0.778	0.778	0.889	0.889	0.889	0.889	0.833	0.889	0.778	0.889	0.833	0.833	0.944	1.000	0.889	1.000	0.833	0.889	0.889	0.889	0.364	0.889	0.833	1.000	0.778	0.833	0.944	0.778	0.889	0.889	0.889	1.000	0.889	0.944	1.000
**103**	0.889	1.000	0.889	0.944	0.778	0.833	0.722	0.778	0.778	0.944	0.889	0.722	0.778	0.611	0.778	0.889	0.833	0.778	0.778	0.778	0.944	0.889	0.364	0.889	0.944	0.833	1.000	0.778	0.833	0.722	0.889	0.833	0.778	0.778	0.889	0.889	0.889	0.889	0.833	0.889	0.778	0.889	0.833	0.833	0.944	1.000	0.889	1.000	0.833	0.889	0.889	0.889	0.364	0.889	0.833	1.000	0.778	0.833	0.944	0.778	0.889	0.889	0.889	1.000	0.889	0.944	1.000	1.000
**104**	0.944	0.944	0.944	1.000	0.722	0.778	0.778	0.833	0.833	0.889	0.944	0.667	0.833	0.667	0.833	0.833	0.889	0.833	0.833	0.833	0.889	0.833	0.364	0.944	0.889	0.778	0.944	0.833	0.889	0.778	0.833	0.889	0.833	0.722	0.944	0.944	0.944	0.833	0.889	0.833	0.722	0.833	0.889	0.889	0.889	0.944	0.944	0.944	0.889	0.944	0.944	0.944	0.364	0.833	0.889	0.944	0.833	0.889	1.000	0.833	0.944	0.833	0.944	0.944	0.944	0.889	0.944	0.944	1.000
**105**	0.364	0.364	0.364	0.364	0.364	0.273	0.273	0.364	0.364	0.364	0.364	0.364	0.364	0.364	0.364	0.364	0.364	0.273	0.364	0.364	0.364	0.364	1.000	0.364	0.364	0.364	0.364	0.364	0.364	0.273	0.364	0.364	0.364	0.364	0.364	0.364	0.364	0.364	0.364	0.364	0.364	0.364	0.364	0.364	0.364	0.364	0.364	0.364	0.364	0.364	0.364	0.364	1.000	0.364	0.364	0.364	0.364	0.364	0.364	0.364	0.364	0.364	0.364	0.364	0.364	0.364	0.364	0.364	0.364	1.000
**134**	0.889	0.889	0.889	0.833	0.778	0.833	0.722	0.778	0.778	0.944	0.889	0.722	0.778	0.611	0.778	0.778	0.722	0.667	0.778	0.778	0.833	0.889	0.364	0.778	0.833	0.833	0.889	0.778	0.722	0.722	0.889	0.833	0.778	0.778	0.778	0.778	0.778	0.778	0.722	0.889	0.778	0.889	0.944	0.833	0.944	0.889	0.889	0.889	0.833	0.889	0.889	0.889	0.364	0.778	0.722	0.889	0.778	0.833	0.833	0.889	0.889	0.889	0.889	0.889	0.889	0.833	0.889	0.889	0.833	0.364	1.000
**146**	0.833	0.833	0.833	0.889	0.833	0.667	0.778	0.944	0.833	0.778	0.833	0.667	0.833	0.778	0.833	0.833	0.889	0.833	0.833	0.833	0.778	0.833	0.364	0.833	0.889	0.889	0.833	0.833	0.889	0.778	0.833	0.778	0.833	0.833	0.944	0.944	0.944	0.833	1.000	0.833	0.833	0.833	0.778	0.778	0.778	0.833	0.833	0.833	0.889	0.833	0.833	0.833	0.364	0.833	0.889	0.833	0.833	0.889	0.889	0.833	0.833	0.833	0.833	0.833	0.833	0.889	0.833	0.833	0.889	0.364	0.722	1.000
**152**	1.000	0.889	1.000	0.944	0.778	0.833	0.833	0.889	0.889	0.944	1.000	0.722	0.889	0.722	0.889	0.778	0.833	0.778	0.889	0.889	0.833	0.889	0.364	0.889	0.833	0.833	0.889	0.889	0.833	0.833	0.889	0.944	0.889	0.778	0.889	0.889	0.889	0.778	0.833	0.889	0.778	0.889	0.944	0.833	0.944	0.889	1.000	0.889	0.944	1.000	1.000	1.000	0.364	0.778	0.833	0.889	0.889	0.944	0.944	0.889	1.000	0.889	1.000	0.889	1.000	0.833	0.889	0.889	0.944	0.364	0.889	0.833	1.000
**154**	0.778	0.889	0.778	0.833	0.889	0.833	0.722	0.778	0.778	0.833	0.778	0.722	0.778	0.722	0.778	0.889	0.833	0.778	0.778	0.778	0.833	0.778	0.364	0.889	0.944	0.833	0.889	0.778	0.833	0.722	0.778	0.833	0.778	0.889	0.889	0.778	0.778	0.889	0.833	0.778	0.889	0.778	0.722	0.722	0.833	0.889	0.778	0.889	0.833	0.778	0.778	0.778	0.364	0.889	0.833	0.889	0.889	0.722	0.833	0.667	0.778	0.889	0.778	0.889	0.778	0.944	0.889	0.889	0.833	0.364	0.778	0.833	0.778	1.000
**155**	0.889	0.889	0.889	0.944	0.667	0.722	0.722	0.778	0.778	0.833	0.889	0.722	0.778	0.722	0.778	0.889	0.944	0.889	0.889	0.778	0.833	0.778	0.364	0.889	0.833	0.722	0.889	0.889	0.944	0.722	0.778	0.833	0.778	0.667	0.889	0.889	0.889	0.778	0.833	0.778	0.667	0.778	0.833	0.833	0.833	0.889	0.889	0.889	0.833	0.889	0.889	0.889	0.364	0.778	0.833	0.889	0.778	0.833	0.944	0.778	0.889	0.778	0.889	0.889	0.889	0.833	0.889	0.889	0.944	0.364	0.778	0.833	0.889	0.778	1.000
**156**	1.000	0.889	1.000	0.944	0.778	0.833	0.833	0.889	0.889	0.944	1.000	0.722	0.889	0.722	0.889	0.778	0.833	0.778	0.889	0.889	0.833	0.889	0.364	0.889	0.833	0.833	0.889	0.889	0.833	0.833	0.889	0.944	0.889	0.778	0.889	0.889	0.889	0.778	0.833	0.889	0.778	0.889	0.944	0.833	0.944	0.889	1.000	0.889	0.944	1.000	1.000	1.000	0.364	0.778	0.833	0.889	0.889	0.944	0.944	0.889	1.000	0.889	1.000	0.889	1.000	0.833	0.889	0.889	0.944	0.364	0.889	0.833	1.000	0.778	0.889	1.000
**157**	0.944	0.944	0.944	1.000	0.722	0.778	0.778	0.833	0.833	0.889	0.944	0.667	0.833	0.667	0.833	0.833	0.889	0.833	0.833	0.833	0.889	0.833	0.364	0.944	0.889	0.778	0.944	0.833	0.889	0.778	0.833	0.889	0.833	0.722	0.944	0.944	0.944	0.833	0.889	0.833	0.722	0.833	0.889	0.889	0.889	0.944	0.944	0.944	0.889	0.944	0.944	0.944	0.364	0.833	0.889	0.944	0.833	0.889	1.000	0.833	0.944	0.833	0.944	0.944	0.944	0.889	0.944	0.944	1.000	0.364	0.833	0.889	0.944	0.833	0.944	0.944	1.000
**158**	0.889	0.778	0.889	0.833	0.778	0.722	0.722	0.889	0.778	0.833	0.889	0.722	0.778	0.833	0.778	0.889	0.944	0.889	1.000	0.778	0.722	0.778	0.364	0.778	0.833	0.833	0.778	1.000	0.944	0.722	0.778	0.833	0.778	0.778	0.889	0.778	0.778	0.667	0.833	0.778	0.778	0.778	0.833	0.722	0.833	0.778	0.889	0.778	0.944	0.889	0.889	0.889	0.364	0.667	0.722	0.778	0.889	0.833	0.833	0.778	0.889	0.889	0.889	0.778	0.889	0.833	0.778	0.778	0.833	0.364	0.778	0.833	0.889	0.778	0.889	0.889	0.833	1.000
**159**	0.944	0.944	0.944	0.889	0.833	0.889	0.778	0.833	0.833	1.000	0.944	0.778	0.833	0.667	0.833	0.833	0.778	0.722	0.833	0.833	0.889	0.944	0.364	0.833	0.889	0.889	0.944	0.833	0.778	0.778	0.944	0.889	0.833	0.833	0.833	0.833	0.833	0.833	0.778	0.944	0.833	0.944	0.889	0.778	1.000	0.944	0.944	0.944	0.889	0.944	0.944	0.944	0.364	0.833	0.778	0.944	0.833	0.889	0.889	0.833	0.944	0.944	0.944	0.944	0.944	0.889	0.944	0.944	0.889	0.364	0.944	0.778	0.944	0.833	0.833	0.944	0.889	0.833	1.000
**160**	1.000	0.889	1.000	0.944	0.778	0.833	0.833	0.889	0.889	0.944	1.000	0.722	0.889	0.722	0.889	0.778	0.833	0.778	0.889	0.889	0.833	0.889	0.364	0.889	0.833	0.833	0.889	0.889	0.833	0.833	0.889	0.944	0.889	0.778	0.889	0.889	0.889	0.778	0.833	0.889	0.778	0.889	0.944	0.833	0.944	0.889	1.000	0.889	0.944	1.000	1.000	1.000	0.364	0.778	0.833	0.889	0.889	0.944	0.944	0.889	1.000	0.889	1.000	0.889	1.000	0.833	0.889	0.889	0.944	0.364	0.889	0.833	1.000	0.778	0.889	1.000	0.944	0.889	0.944	1.000
**161**	1.000	0.889	1.000	0.944	0.778	0.833	0.833	0.889	0.889	0.944	1.000	0.722	0.889	0.722	0.889	0.778	0.833	0.778	0.889	0.889	0.833	0.889	0.364	0.889	0.833	0.833	0.889	0.889	0.833	0.833	0.889	0.944	0.889	0.778	0.889	0.889	0.889	0.778	0.833	0.889	0.778	0.889	0.944	0.833	0.944	0.889	1.000	0.889	0.944	1.000	1.000	1.000	0.364	0.778	0.833	0.889	0.889	0.944	0.944	0.889	1.000	0.889	1.000	0.889	1.000	0.833	0.889	0.889	0.944	0.364	0.889	0.833	1.000	0.778	0.889	1.000	0.944	0.889	0.944	1.000	1.000
**162**	0.778	0.889	0.778	0.833	0.667	0.722	0.611	0.667	0.667	0.833	0.778	0.722	0.667	0.611	0.667	0.889	0.833	0.778	0.778	0.667	0.833	0.778	0.364	0.778	0.833	0.722	0.889	0.778	0.833	0.611	0.778	0.722	0.667	0.667	0.778	0.778	0.778	0.778	0.722	0.778	0.667	0.778	0.722	0.722	0.833	0.889	0.778	0.889	0.722	0.778	0.778	0.778	0.364	0.778	0.722	0.889	0.667	0.722	0.833	0.667	0.778	0.778	0.778	0.889	0.778	0.833	0.889	0.889	0.833	0.364	0.778	0.722	0.778	0.778	0.889	0.778	0.833	0.778	0.833	0.778	0.778	1.000
**163**	0.889	0.889	0.889	0.944	0.778	0.722	0.722	0.889	0.778	0.833	0.889	0.611	0.778	0.722	0.778	0.889	0.944	0.889	0.889	0.778	0.833	0.778	0.364	0.889	0.944	0.833	0.889	0.889	0.944	0.722	0.778	0.833	0.778	0.778	1.000	0.889	0.889	0.778	0.944	0.778	0.778	0.778	0.833	0.833	0.833	0.889	0.889	0.889	0.944	0.889	0.889	0.889	0.364	0.778	0.833	0.889	0.889	0.833	0.944	0.778	0.889	0.889	0.889	0.889	0.889	0.944	0.889	0.889	0.944	0.364	0.778	0.944	0.889	0.889	0.889	0.889	0.944	0.889	0.833	0.889	0.889	0.778	1.000
**164**	0.833	0.833	0.833	0.889	0.722	0.667	0.667	0.833	0.722	0.778	0.833	0.667	0.722	0.778	0.722	0.944	1.000	0.944	0.944	0.722	0.778	0.722	0.364	0.833	0.889	0.778	0.833	0.944	1.000	0.667	0.722	0.778	0.722	0.722	0.944	0.833	0.833	0.722	0.889	0.722	0.722	0.722	0.778	0.778	0.778	0.833	0.833	0.833	0.889	0.833	0.833	0.833	0.364	0.722	0.778	0.833	0.833	0.778	0.889	0.722	0.833	0.833	0.833	0.833	0.833	0.889	0.833	0.833	0.889	0.364	0.722	0.889	0.833	0.833	0.944	0.833	0.889	0.944	0.778	0.833	0.833	0.833	0.944	1.000
**165**	0.722	0.611	0.722	0.667	0.833	0.667	0.778	0.833	0.833	0.667	0.722	0.889	0.833	1.000	0.833	0.722	0.778	0.722	0.833	0.833	0.556	0.722	0.364	0.722	0.667	0.778	0.611	0.833	0.778	0.778	0.722	0.778	0.833	0.833	0.722	0.722	0.722	0.722	0.778	0.722	0.833	0.722	0.667	0.556	0.667	0.611	0.722	0.611	0.778	0.722	0.722	0.722	0.364	0.722	0.778	0.611	0.833	0.778	0.667	0.722	0.722	0.722	0.722	0.611	0.722	0.667	0.611	0.611	0.667	0.364	0.611	0.778	0.722	0.722	0.722	0.722	0.667	0.833	0.667	0.722	0.722	0.611	0.722	0.778	1.000
**166**	0.833	0.833	0.833	0.778	0.833	0.778	0.778	0.833	0.833	0.889	0.833	0.889	0.833	0.778	0.833	0.833	0.778	0.722	0.833	0.833	0.778	0.944	0.364	0.722	0.778	0.889	0.833	0.833	0.778	0.778	0.944	0.778	0.833	0.833	0.722	0.833	0.833	0.833	0.778	0.944	0.833	0.944	0.778	0.667	0.889	0.833	0.833	0.833	0.778	0.833	0.833	0.833	0.364	0.833	0.778	0.833	0.722	0.889	0.778	0.833	0.833	0.833	0.833	0.833	0.833	0.778	0.833	0.833	0.778	0.364	0.833	0.778	0.833	0.722	0.833	0.833	0.778	0.833	0.889	0.833	0.833	0.833	0.722	0.778	0.778	1.000
**167**	0.833	0.833	0.833	0.889	0.611	0.667	0.667	0.722	0.722	0.778	0.833	0.556	0.722	0.556	0.722	0.722	0.778	0.722	0.722	0.722	0.889	0.722	0.364	0.833	0.778	0.667	0.833	0.722	0.778	0.667	0.722	0.778	0.722	0.611	0.833	0.833	0.833	0.722	0.778	0.722	0.611	0.722	0.889	1.000	0.778	0.833	0.833	0.833	0.778	0.833	0.833	0.833	0.364	0.722	0.778	0.833	0.722	0.778	0.889	0.833	0.833	0.722	0.833	0.833	0.833	0.778	0.833	0.833	0.889	0.364	0.833	0.778	0.833	0.722	0.833	0.833	0.889	0.722	0.778	0.833	0.833	0.722	0.833	0.778	0.556	0.667	1.000
**168**	0.889	1.000	0.889	0.944	0.778	0.833	0.722	0.778	0.778	0.944	0.889	0.722	0.778	0.611	0.778	0.889	0.833	0.778	0.778	0.778	0.944	0.889	0.364	0.889	0.944	0.833	1.000	0.778	0.833	0.722	0.889	0.833	0.778	0.778	0.889	0.889	0.889	0.889	0.833	0.889	0.778	0.889	0.833	0.833	0.944	1.000	0.889	1.000	0.833	0.889	0.889	0.889	0.364	0.889	0.833	1.000	0.778	0.833	0.944	0.778	0.889	0.889	0.889	1.000	0.889	0.944	1.000	1.000	0.944	0.364	0.889	0.833	0.889	0.889	0.889	0.889	0.944	0.778	0.944	0.889	0.889	0.889	0.889	0.833	0.611	0.833	0.833	1.000
**169**	0.833	0.944	0.833	0.889	0.833	0.778	0.667	0.833	0.722	0.889	0.833	0.667	0.722	0.667	0.722	0.944	0.889	0.833	0.833	0.722	0.889	0.833	0.364	0.833	1.000	0.889	0.944	0.833	0.889	0.667	0.833	0.778	0.722	0.833	0.944	0.833	0.833	0.833	0.889	0.833	0.833	0.833	0.778	0.778	0.889	0.944	0.833	0.944	0.889	0.833	0.833	0.833	0.364	0.833	0.778	0.944	0.833	0.778	0.889	0.722	0.833	0.944	0.833	0.944	0.833	1.000	0.944	0.944	0.889	0.364	0.833	0.889	0.833	0.944	0.833	0.833	0.889	0.833	0.889	0.833	0.833	0.833	0.944	0.889	0.667	0.778	0.778	0.944	1.000
**170**	0.833	0.944	0.833	0.889	0.833	0.778	0.667	0.833	0.722	0.889	0.833	0.667	0.722	0.667	0.722	0.944	0.889	0.833	0.833	0.722	0.889	0.833	0.364	0.833	1.000	0.889	0.944	0.833	0.889	0.667	0.833	0.778	0.722	0.833	0.944	0.833	0.833	0.833	0.889	0.833	0.833	0.833	0.778	0.778	0.889	0.944	0.833	0.944	0.889	0.833	0.833	0.833	0.364	0.833	0.778	0.944	0.833	0.778	0.889	0.722	0.833	0.944	0.833	0.944	0.833	1.000	0.944	0.944	0.889	0.364	0.833	0.889	0.833	0.944	0.833	0.833	0.889	0.833	0.889	0.833	0.833	0.833	0.944	0.889	0.667	0.778	0.778	0.944	1.000	1.000
