# Supplementary material for: Assessment of Wild Rocket (Diplotaxis tenuifolia (L.) DC.) Germplasm Accessions by NGS Identified SSR and SNP Markers
Source: Plants (Basel). 2022 Dec 12;11(24):3482. doi: 10.3390/plants11243482 (PMC9783343; doi:10.3390/plants11243482)
Supplement: Supplementary file 1 [file Table S6.pdf › Table S6.pdf]

Table SM6. Genetic similarity matrix (SSR + SNP-CAPS markers)

Accessions	1	3	4	5	6	7	8	9	10	12	15	16	17	18	19	20	21	22	23	24	26	27	29	32	35	36	37	38	39	40	41	42	43	44	45	46	47	48	49	51	52	54	55	56	57	58	59	60	61	63	64	65	68	69	72	73	74	81	84	91	92	97	98	99	100	101	102	103	104	105	134	146	152	154	155	156	157	158	159	160	161	162	163	164	165	166	167	168	169	170
**1**	1.000
**3**	0.893	1.000
**4**	0.964	0.857	1.000
**5**	0.821	0.786	0.857	1.000
**6**	0.750	0.643	0.786	0.786	1.000
**7**	0.821	0.714	0.857	0.821	0.857	1.000
**8**	0.714	0.607	0.714	0.679	0.750	0.786	1.000
**9**	0.821	0.714	0.857	0.750	0.857	0.786	0.714	1.000
**10**	0.786	0.679	0.750	0.750	0.786	0.750	0.857	0.750	1.000
**12**	0.893	0.786	0.929	0.857	0.750	0.821	0.679	0.857	0.714	1.000
**15**	0.964	0.857	0.929	0.786	0.786	0.786	0.679	0.857	0.750	0.857	1.000
**16**	0.679	0.643	0.643	0.643	0.679	0.643	0.750	0.571	0.821	0.571	0.643	1.000
**17**	0.714	0.607	0.714	0.714	0.786	0.750	0.929	0.750	0.857	0.714	0.679	0.750	1.000
**18**	0.679	0.571	0.643	0.607	0.750	0.643	0.750	0.643	0.786	0.571	0.714	0.893	0.750	1.000
**19**	0.786	0.679	0.750	0.750	0.786	0.750	0.857	0.750	1.000	0.714	0.750	0.821	0.857	0.786	1.000
**20**	0.786	0.821	0.821	0.750	0.679	0.679	0.571	0.750	0.571	0.750	0.821	0.643	0.571	0.643	0.571	1.000
**21**	0.679	0.643	0.714	0.786	0.679	0.643	0.607	0.750	0.643	0.714	0.714	0.571	0.643	0.607	0.643	0.786	1.000
**22**	0.607	0.571	0.607	0.679	0.571	0.607	0.607	0.643	0.571	0.607	0.643	0.500	0.571	0.536	0.571	0.679	0.893	1.000
**23**	0.679	0.571	0.679	0.714	0.679	0.643	0.643	0.714	0.714	0.679	0.714	0.643	0.679	0.679	0.714	0.679	0.821	0.786	1.000
**24**	0.714	0.607	0.714	0.714	0.786	0.750	0.929	0.750	0.857	0.714	0.679	0.750	1.000	0.750	0.857	0.571	0.643	0.571	0.679	1.000
**26**	0.714	0.750	0.750	0.786	0.679	0.714	0.536	0.679	0.607	0.714	0.679	0.571	0.571	0.500	0.607	0.714	0.643	0.536	0.571	0.571	1.000
**27**	0.750	0.714	0.786	0.786	0.857	0.786	0.750	0.857	0.786	0.786	0.714	0.679	0.786	0.607	0.786	0.679	0.679	0.571	0.679	0.786	0.750	1.000
**29**	0.162	0.162	0.162	0.216	0.216	0.162	0.216	0.216	0.270	0.162	0.162	0.216	0.270	0.216	0.270	0.162	0.216	0.162	0.270	0.270	0.162	0.270	1.000
**32**	0.821	0.786	0.857	0.893	0.786	0.821	0.714	0.714	0.786	0.786	0.786	0.679	0.714	0.643	0.786	0.786	0.750	0.643	0.643	0.714	0.750	0.714	0.162	1.000
**35**	0.821	0.857	0.786	0.786	0.679	0.643	0.607	0.714	0.679	0.714	0.857	0.679	0.607	0.643	0.679	0.857	0.750	0.679	0.679	0.607	0.714	0.679	0.162	0.786	1.000
**36**	0.786	0.750	0.750	0.643	0.750	0.607	0.643	0.750	0.679	0.679	0.821	0.679	0.643	0.679	0.679	0.750	0.643	0.643	0.607	0.643	0.607	0.750	0.162	0.643	0.821	1.000
**37**	0.893	0.929	0.929	0.857	0.714	0.786	0.643	0.786	0.679	0.857	0.857	0.643	0.643	0.571	0.679	0.893	0.714	0.607	0.607	0.643	0.821	0.786	0.162	0.857	0.857	0.750	1.000
**38**	0.679	0.571	0.679	0.714	0.679	0.643	0.643	0.714	0.714	0.679	0.714	0.643	0.679	0.679	0.714	0.679	0.821	0.786	1.000	0.679	0.571	0.679	0.270	0.643	0.679	0.607	0.607	1.000
**39**	0.679	0.643	0.679	0.750	0.643	0.607	0.607	0.714	0.643	0.679	0.714	0.571	0.643	0.607	0.643	0.750	0.964	0.929	0.857	0.643	0.607	0.643	0.216	0.714	0.750	0.643	0.679	0.857	1.000
**40**	0.750	0.643	0.750	0.679	0.750	0.821	0.893	0.786	0.786	0.750	0.714	0.643	0.893	0.643	0.786	0.607	0.607	0.607	0.643	0.893	0.536	0.750	0.216	0.679	0.607	0.643	0.679	0.643	0.607	1.000
**41**	0.786	0.750	0.821	0.750	0.857	0.786	0.679	0.893	0.679	0.786	0.821	0.679	0.714	0.679	0.679	0.821	0.679	0.571	0.679	0.714	0.714	0.857	0.216	0.679	0.786	0.821	0.821	0.679	0.643	0.750	1.000
**42**	0.821	0.714	0.821	0.821	0.857	0.821	0.750	0.750	0.821	0.750	0.857	0.714	0.750	0.750	0.821	0.714	0.714	0.643	0.750	0.750	0.643	0.714	0.216	0.857	0.786	0.714	0.750	0.750	0.714	0.714	0.750	1.000
**43**	0.786	0.679	0.821	0.821	0.929	0.857	0.786	0.786	0.821	0.786	0.750	0.714	0.821	0.714	0.821	0.643	0.643	0.536	0.643	0.821	0.679	0.857	0.216	0.821	0.643	0.714	0.750	0.643	0.607	0.786	0.786	0.857	1.000
**44**	0.679	0.643	0.679	0.643	0.821	0.679	0.714	0.714	0.750	0.679	0.714	0.679	0.714	0.679	0.750	0.643	0.571	0.571	0.607	0.714	0.571	0.786	0.216	0.679	0.679	0.821	0.679	0.607	0.571	0.679	0.750	0.786	0.786	1.000
**45**	0.857	0.821	0.857	0.857	0.786	0.786	0.679	0.786	0.714	0.786	0.893	0.607	0.679	0.679	0.714	0.821	0.750	0.679	0.714	0.679	0.714	0.714	0.216	0.821	0.821	0.714	0.857	0.714	0.750	0.679	0.786	0.893	0.786	0.714	1.000
**46**	0.821	0.714	0.857	0.786	0.821	0.821	0.786	0.929	0.786	0.857	0.786	0.643	0.821	0.643	0.786	0.714	0.714	0.607	0.714	0.821	0.679	0.893	0.270	0.714	0.679	0.714	0.786	0.714	0.679	0.857	0.893	0.714	0.821	0.679	0.750	1.000
**47**	0.786	0.679	0.821	0.750	0.750	0.750	0.714	0.857	0.750	0.821	0.750	0.571	0.750	0.571	0.750	0.643	0.679	0.643	0.643	0.750	0.643	0.821	0.216	0.714	0.607	0.714	0.750	0.643	0.643	0.750	0.750	0.714	0.821	0.714	0.750	0.857	1.000
**48**	0.750	0.786	0.786	0.821	0.821	0.786	0.679	0.786	0.750	0.750	0.714	0.714	0.714	0.607	0.750	0.750	0.679	0.571	0.571	0.714	0.786	0.821	0.162	0.857	0.786	0.714	0.857	0.571	0.643	0.714	0.821	0.750	0.821	0.714	0.714	0.786	0.714	1.000
**49**	0.750	0.714	0.786	0.857	0.821	0.714	0.679	0.821	0.714	0.750	0.786	0.643	0.714	0.679	0.714	0.786	0.786	0.679	0.714	0.714	0.679	0.750	0.216	0.786	0.821	0.750	0.786	0.714	0.750	0.714	0.857	0.821	0.821	0.679	0.857	0.821	0.750	0.821	1.000
**51**	0.821	0.786	0.857	0.786	0.750	0.750	0.679	0.857	0.714	0.929	0.786	0.643	0.714	0.571	0.714	0.750	0.643	0.536	0.607	0.714	0.714	0.857	0.162	0.714	0.714	0.750	0.857	0.607	0.607	0.750	0.857	0.679	0.786	0.750	0.714	0.857	0.821	0.821	0.750	1.000
**52**	0.679	0.643	0.714	0.714	0.857	0.750	0.643	0.786	0.750	0.679	0.714	0.714	0.679	0.679	0.750	0.679	0.643	0.536	0.679	0.679	0.750	0.786	0.162	0.750	0.714	0.714	0.714	0.679	0.607	0.643	0.821	0.786	0.786	0.750	0.679	0.714	0.679	0.857	0.750	0.750	1.000
**54**	0.714	0.679	0.750	0.714	0.786	0.679	0.643	0.821	0.679	0.750	0.750	0.607	0.679	0.607	0.679	0.714	0.679	0.571	0.643	0.679	0.714	0.821	0.162	0.679	0.714	0.750	0.750	0.643	0.643	0.643	0.821	0.750	0.786	0.750	0.750	0.750	0.821	0.750	0.786	0.821	0.786	1.000
**55**	0.750	0.714	0.786	0.750	0.679	0.714	0.679	0.714	0.679	0.786	0.714	0.571	0.714	0.571	0.679	0.679	0.714	0.607	0.679	0.714	0.643	0.714	0.162	0.750	0.643	0.607	0.714	0.679	0.679	0.643	0.679	0.679	0.714	0.571	0.679	0.750	0.750	0.643	0.679	0.714	0.607	0.750	1.000
**56**	0.741	0.778	0.741	0.741	0.593	0.630	0.630	0.704	0.630	0.741	0.704	0.481	0.667	0.481	0.630	0.667	0.704	0.630	0.667	0.667	0.704	0.667	0.229	0.704	0.741	0.593	0.741	0.667	0.704	0.667	0.630	0.630	0.630	0.519	0.704	0.741	0.667	0.667	0.704	0.667	0.556	0.593	0.741	1.000
**57**	0.750	0.714	0.786	0.750	0.750	0.750	0.714	0.821	0.679	0.786	0.786	0.607	0.750	0.607	0.679	0.786	0.750	0.643	0.750	0.750	0.679	0.821	0.270	0.679	0.750	0.714	0.786	0.750	0.714	0.750	0.857	0.750	0.714	0.714	0.786	0.821	0.786	0.679	0.750	0.786	0.714	0.821	0.750	0.667	1.000
**58**	0.750	0.786	0.786	0.821	0.643	0.679	0.607	0.750	0.679	0.786	0.714	0.571	0.643	0.464	0.679	0.750	0.750	0.714	0.643	0.643	0.750	0.786	0.216	0.786	0.786	0.714	0.857	0.643	0.714	0.643	0.714	0.714	0.714	0.679	0.750	0.750	0.821	0.821	0.786	0.786	0.679	0.750	0.679	0.741	0.750	1.000
**59**	0.821	0.714	0.857	0.929	0.786	0.821	0.750	0.821	0.821	0.893	0.786	0.643	0.786	0.607	0.821	0.679	0.786	0.679	0.786	0.786	0.714	0.857	0.270	0.821	0.714	0.643	0.786	0.786	0.750	0.750	0.750	0.821	0.821	0.679	0.786	0.857	0.821	0.750	0.786	0.821	0.714	0.750	0.786	0.741	0.821	0.821	1.000
**60**	0.750	0.714	0.786	0.893	0.750	0.786	0.714	0.786	0.750	0.786	0.714	0.607	0.750	0.571	0.750	0.714	0.857	0.750	0.750	0.750	0.750	0.821	0.270	0.821	0.750	0.643	0.786	0.750	0.821	0.714	0.750	0.750	0.750	0.607	0.786	0.857	0.750	0.786	0.821	0.714	0.679	0.679	0.750	0.778	0.786	0.821	0.893	1.000
**61**	0.786	0.679	0.821	0.857	0.821	0.786	0.714	0.857	0.786	0.821	0.821	0.607	0.750	0.643	0.786	0.714	0.821	0.714	0.821	0.750	0.679	0.821	0.270	0.786	0.750	0.679	0.750	0.821	0.786	0.714	0.786	0.893	0.821	0.714	0.857	0.821	0.821	0.714	0.857	0.750	0.750	0.821	0.750	0.704	0.857	0.821	0.929	0.857	1.000
**63**	0.857	0.750	0.893	0.857	0.786	0.857	0.714	0.821	0.750	0.893	0.821	0.607	0.750	0.607	0.750	0.714	0.714	0.607	0.714	0.750	0.714	0.821	0.216	0.786	0.679	0.643	0.821	0.714	0.679	0.750	0.786	0.821	0.857	0.679	0.857	0.857	0.857	0.714	0.786	0.821	0.679	0.821	0.821	0.704	0.821	0.786	0.893	0.821	0.893	1.000
**64**	0.821	0.714	0.857	0.821	0.821	0.821	0.679	0.786	0.679	0.821	0.786	0.607	0.714	0.607	0.679	0.714	0.714	0.607	0.679	0.714	0.750	0.821	0.162	0.786	0.714	0.714	0.786	0.679	0.679	0.679	0.786	0.750	0.821	0.643	0.750	0.821	0.786	0.750	0.750	0.750	0.714	0.750	0.821	0.704	0.786	0.714	0.821	0.821	0.786	0.821	1.000
**65**	0.857	0.750	0.893	0.821	0.786	0.857	0.714	0.893	0.750	0.893	0.821	0.571	0.750	0.571	0.750	0.714	0.786	0.679	0.714	0.750	0.750	0.857	0.216	0.786	0.679	0.679	0.821	0.714	0.750	0.750	0.786	0.750	0.786	0.643	0.786	0.893	0.857	0.750	0.714	0.821	0.714	0.750	0.786	0.741	0.821	0.786	0.893	0.893	0.857	0.893	0.857	1.000
**68**	0.229	0.229	0.229	0.229	0.229	0.171	0.171	0.229	0.229	0.229	0.229	0.229	0.229	0.229	0.229	0.229	0.229	0.171	0.229	0.229	0.229	0.229	0.375	0.229	0.229	0.229	0.229	0.229	0.229	0.171	0.229	0.229	0.229	0.229	0.229	0.229	0.229	0.229	0.229	0.229	0.229	0.229	0.229	0.242	0.229	0.229	0.229	0.229	0.229	0.229	0.229	0.229	1.000
**69**	0.679	0.714	0.679	0.750	0.714	0.679	0.786	0.679	0.821	0.643	0.643	0.750	0.786	0.643	0.821	0.679	0.714	0.643	0.714	0.786	0.643	0.786	0.270	0.786	0.750	0.679	0.750	0.714	0.714	0.750	0.714	0.750	0.750	0.750	0.714	0.750	0.679	0.821	0.750	0.714	0.714	0.679	0.643	0.667	0.714	0.750	0.750	0.786	0.714	0.679	0.643	0.679	0.229	1.000
**72**	0.821	0.786	0.786	0.786	0.786	0.786	0.821	0.714	0.821	0.714	0.786	0.786	0.821	0.786	0.821	0.714	0.643	0.571	0.607	0.821	0.643	0.714	0.216	0.821	0.750	0.714	0.786	0.607	0.643	0.786	0.750	0.786	0.821	0.714	0.821	0.786	0.714	0.786	0.786	0.714	0.679	0.643	0.643	0.667	0.679	0.643	0.714	0.750	0.679	0.750	0.714	0.714	0.229	0.821	1.000
**73**	0.786	0.821	0.786	0.821	0.643	0.714	0.607	0.714	0.643	0.857	0.750	0.571	0.643	0.500	0.643	0.750	0.679	0.607	0.607	0.643	0.750	0.750	0.162	0.750	0.750	0.643	0.857	0.607	0.679	0.643	0.714	0.679	0.679	0.679	0.786	0.714	0.750	0.750	0.679	0.857	0.643	0.750	0.714	0.704	0.786	0.786	0.786	0.750	0.714	0.786	0.750	0.786	0.229	0.714	0.714	1.000
**74**	0.786	0.679	0.821	0.786	0.893	0.893	0.750	0.821	0.714	0.786	0.821	0.679	0.786	0.750	0.714	0.750	0.714	0.607	0.714	0.786	0.679	0.750	0.216	0.786	0.714	0.679	0.750	0.714	0.679	0.750	0.857	0.857	0.821	0.714	0.821	0.821	0.714	0.750	0.786	0.714	0.786	0.714	0.714	0.630	0.821	0.643	0.786	0.786	0.821	0.821	0.821	0.821	0.229	0.679	0.821	0.679	1.000
**81**	0.857	0.750	0.893	0.857	0.857	0.857	0.750	0.857	0.786	0.857	0.821	0.679	0.786	0.679	0.786	0.714	0.679	0.571	0.679	0.786	0.714	0.857	0.216	0.786	0.679	0.714	0.821	0.679	0.643	0.786	0.857	0.821	0.929	0.714	0.857	0.893	0.893	0.786	0.857	0.857	0.750	0.821	0.750	0.667	0.786	0.750	0.857	0.786	0.857	0.929	0.821	0.857	0.229	0.714	0.821	0.750	0.821	1.000
**84**	0.857	0.821	0.893	0.893	0.750	0.821	0.643	0.786	0.679	0.857	0.821	0.571	0.679	0.571	0.679	0.786	0.750	0.643	0.643	0.679	0.786	0.750	0.162	0.857	0.750	0.643	0.893	0.643	0.714	0.679	0.750	0.786	0.821	0.607	0.893	0.786	0.857	0.786	0.821	0.786	0.679	0.786	0.786	0.741	0.786	0.821	0.821	0.821	0.821	0.893	0.857	0.857	0.229	0.679	0.786	0.857	0.786	0.893	1.000
**91**	0.852	0.815	0.852	0.778	0.778	0.778	0.741	0.815	0.741	0.815	0.815	0.667	0.778	0.667	0.741	0.704	0.630	0.556	0.667	0.778	0.630	0.778	0.229	0.704	0.704	0.741	0.778	0.667	0.630	0.815	0.852	0.741	0.815	0.667	0.778	0.889	0.778	0.741	0.815	0.815	0.667	0.704	0.778	0.769	0.741	0.667	0.778	0.741	0.741	0.815	0.778	0.778	0.242	0.704	0.815	0.704	0.778	0.889	0.778	1.000
**92**	0.778	0.667	0.778	0.815	0.704	0.741	0.704	0.704	0.778	0.815	0.741	0.667	0.741	0.630	0.778	0.630	0.704	0.630	0.815	0.741	0.667	0.741	0.229	0.741	0.704	0.630	0.704	0.815	0.704	0.704	0.704	0.778	0.741	0.667	0.741	0.778	0.741	0.667	0.741	0.741	0.704	0.704	0.778	0.731	0.778	0.704	0.852	0.815	0.815	0.815	0.815	0.778	0.242	0.704	0.704	0.778	0.741	0.778	0.778	0.769	1.000
**97**	0.741	0.704	0.741	0.667	0.667	0.630	0.630	0.741	0.630	0.778	0.778	0.593	0.667	0.593	0.630	0.778	0.667	0.593	0.667	0.667	0.630	0.704	0.171	0.667	0.778	0.741	0.741	0.667	0.667	0.630	0.778	0.704	0.630	0.704	0.704	0.704	0.630	0.667	0.704	0.778	0.667	0.741	0.741	0.654	0.778	0.704	0.704	0.667	0.741	0.704	0.704	0.704	0.242	0.630	0.593	0.741	0.704	0.667	0.667	0.692	0.731	1.000
**98**	1.000	0.893	0.964	0.821	0.750	0.821	0.714	0.821	0.786	0.893	0.964	0.679	0.714	0.679	0.786	0.786	0.679	0.607	0.679	0.714	0.714	0.750	0.162	0.821	0.821	0.786	0.893	0.679	0.679	0.750	0.786	0.821	0.786	0.679	0.857	0.821	0.786	0.750	0.750	0.821	0.679	0.714	0.750	0.741	0.750	0.750	0.821	0.750	0.786	0.857	0.821	0.857	0.229	0.679	0.821	0.786	0.786	0.857	0.857	0.852	0.778	0.741	1.000
**99**	0.929	0.964	0.893	0.821	0.679	0.750	0.643	0.750	0.714	0.821	0.893	0.679	0.643	0.607	0.714	0.857	0.679	0.607	0.607	0.643	0.786	0.750	0.162	0.821	0.893	0.786	0.964	0.607	0.679	0.679	0.786	0.750	0.714	0.679	0.857	0.750	0.714	0.821	0.750	0.821	0.679	0.714	0.679	0.741	0.750	0.821	0.750	0.750	0.714	0.786	0.750	0.786	0.229	0.750	0.821	0.857	0.714	0.786	0.857	0.778	0.704	0.741	0.929	1.000
**100**	0.929	0.893	0.893	0.750	0.750	0.750	0.643	0.821	0.714	0.821	0.964	0.679	0.643	0.679	0.714	0.857	0.679	0.607	0.679	0.643	0.714	0.750	0.162	0.750	0.893	0.857	0.893	0.679	0.679	0.679	0.857	0.821	0.714	0.750	0.857	0.750	0.714	0.750	0.750	0.821	0.750	0.786	0.679	0.667	0.821	0.750	0.750	0.679	0.786	0.786	0.750	0.786	0.229	0.679	0.750	0.786	0.786	0.786	0.786	0.778	0.704	0.815	0.929	0.929	1.000
**101**	0.821	0.857	0.786	0.714	0.643	0.643	0.571	0.714	0.607	0.714	0.857	0.607	0.571	0.607	0.607	0.857	0.679	0.607	0.607	0.571	0.750	0.643	0.162	0.714	0.929	0.786	0.857	0.607	0.679	0.607	0.786	0.714	0.607	0.643	0.821	0.679	0.607	0.714	0.750	0.714	0.643	0.679	0.607	0.778	0.750	0.714	0.643	0.679	0.679	0.679	0.679	0.679	0.229	0.679	0.750	0.750	0.714	0.679	0.750	0.704	0.630	0.741	0.821	0.893	0.893	1.000
**102**	0.786	0.821	0.821	0.821	0.786	0.714	0.607	0.750	0.643	0.750	0.821	0.607	0.607	0.607	0.643	0.857	0.750	0.643	0.643	0.607	0.821	0.786	0.162	0.821	0.857	0.786	0.893	0.643	0.714	0.571	0.786	0.786	0.750	0.750	0.857	0.679	0.679	0.786	0.786	0.750	0.786	0.786	0.679	0.667	0.786	0.786	0.750	0.750	0.786	0.750	0.786	0.750	0.229	0.714	0.714	0.786	0.750	0.750	0.821	0.667	0.667	0.741	0.786	0.857	0.857	0.821	1.000
**103**	0.821	0.857	0.857	0.893	0.786	0.857	0.643	0.786	0.679	0.821	0.786	0.643	0.679	0.571	0.679	0.821	0.714	0.607	0.643	0.679	0.857	0.857	0.216	0.821	0.786	0.679	0.929	0.643	0.679	0.679	0.857	0.750	0.786	0.679	0.857	0.821	0.750	0.857	0.786	0.821	0.750	0.750	0.714	0.704	0.821	0.821	0.821	0.857	0.786	0.857	0.821	0.857	0.229	0.750	0.786	0.857	0.821	0.857	0.893	0.778	0.741	0.704	0.821	0.893	0.821	0.786	0.857	1.000
**104**	0.714	0.750	0.750	0.821	0.750	0.750	0.679	0.750	0.679	0.750	0.679	0.536	0.714	0.536	0.679	0.714	0.750	0.643	0.679	0.714	0.714	0.821	0.270	0.786	0.679	0.607	0.750	0.679	0.714	0.679	0.714	0.679	0.750	0.607	0.750	0.821	0.714	0.714	0.750	0.679	0.607	0.643	0.821	0.815	0.750	0.750	0.821	0.893	0.786	0.786	0.821	0.821	0.229	0.714	0.714	0.714	0.750	0.750	0.786	0.778	0.741	0.667	0.714	0.714	0.643	0.643	0.750	0.821	1.000
**105**	0.167	0.167	0.167	0.167	0.167	0.111	0.111	0.167	0.167	0.167	0.167	0.167	0.167	0.167	0.167	0.167	0.167	0.111	0.167	0.167	0.167	0.167	0.471	0.167	0.167	0.167	0.167	0.167	0.167	0.111	0.167	0.167	0.167	0.167	0.167	0.167	0.167	0.167	0.167	0.167	0.167	0.167	0.167	0.176	0.167	0.167	0.167	0.167	0.167	0.167	0.167	0.167	0.667	0.167	0.167	0.167	0.167	0.167	0.167	0.176	0.176	0.176	0.167	0.167	0.167	0.167	0.167	0.167	0.167	1.000
**134**	0.778	0.815	0.778	0.778	0.667	0.704	0.704	0.741	0.741	0.778	0.741	0.667	0.741	0.556	0.741	0.704	0.704	0.630	0.741	0.741	0.667	0.815	0.286	0.704	0.778	0.704	0.778	0.741	0.704	0.741	0.778	0.741	0.704	0.667	0.704	0.815	0.704	0.741	0.741	0.778	0.667	0.704	0.778	0.808	0.815	0.815	0.852	0.815	0.815	0.778	0.741	0.778	0.242	0.778	0.667	0.741	0.704	0.741	0.704	0.846	0.808	0.769	0.778	0.778	0.778	0.704	0.704	0.778	0.815	0.176	1.000
**146**	0.704	0.667	0.704	0.741	0.741	0.630	0.667	0.778	0.704	0.704	0.741	0.593	0.704	0.667	0.704	0.704	0.704	0.630	0.704	0.704	0.704	0.704	0.229	0.667	0.741	0.704	0.704	0.704	0.704	0.704	0.778	0.704	0.704	0.667	0.778	0.778	0.667	0.704	0.852	0.704	0.667	0.741	0.630	0.692	0.704	0.667	0.704	0.741	0.741	0.704	0.667	0.667	0.242	0.704	0.741	0.667	0.704	0.741	0.704	0.769	0.731	0.692	0.704	0.704	0.704	0.704	0.704	0.704	0.704	0.176	0.692	1.000
**152**	0.929	0.821	0.893	0.821	0.750	0.821	0.786	0.750	0.786	0.821	0.893	0.750	0.786	0.750	0.786	0.750	0.679	0.607	0.714	0.786	0.679	0.750	0.216	0.786	0.786	0.750	0.821	0.714	0.679	0.750	0.786	0.821	0.786	0.679	0.857	0.821	0.750	0.679	0.750	0.750	0.643	0.679	0.750	0.704	0.786	0.679	0.821	0.786	0.786	0.857	0.821	0.821	0.229	0.714	0.893	0.750	0.857	0.857	0.821	0.852	0.815	0.704	0.929	0.857	0.857	0.786	0.750	0.821	0.750	0.167	0.778	0.704	1.000
**154**	0.714	0.750	0.750	0.750	0.750	0.750	0.643	0.750	0.643	0.786	0.750	0.607	0.679	0.607	0.643	0.821	0.714	0.607	0.643	0.679	0.679	0.714	0.216	0.750	0.786	0.679	0.821	0.643	0.679	0.714	0.821	0.750	0.714	0.786	0.786	0.750	0.643	0.786	0.786	0.786	0.714	0.714	0.643	0.667	0.821	0.750	0.714	0.750	0.750	0.750	0.679	0.714	0.229	0.750	0.750	0.786	0.821	0.714	0.750	0.704	0.704	0.778	0.714	0.786	0.786	0.786	0.786	0.821	0.714	0.167	0.741	0.741	0.714	1.000
**155**	0.786	0.750	0.750	0.750	0.714	0.643	0.643	0.750	0.714	0.714	0.821	0.607	0.643	0.679	0.714	0.786	0.821	0.750	0.750	0.643	0.679	0.714	0.216	0.750	0.786	0.714	0.750	0.750	0.821	0.607	0.643	0.750	0.679	0.679	0.821	0.679	0.679	0.643	0.714	0.643	0.643	0.679	0.643	0.704	0.714	0.714	0.750	0.750	0.786	0.714	0.714	0.750	0.229	0.679	0.714	0.714	0.679	0.679	0.750	0.630	0.667	0.667	0.786	0.786	0.786	0.750	0.857	0.714	0.750	0.167	0.667	0.704	0.750	0.679	1.000
**156**	0.857	0.750	0.893	0.857	0.786	0.821	0.679	0.821	0.714	0.857	0.821	0.643	0.714	0.607	0.714	0.786	0.750	0.643	0.714	0.714	0.750	0.786	0.162	0.857	0.786	0.714	0.821	0.714	0.714	0.714	0.821	0.786	0.786	0.607	0.750	0.857	0.750	0.821	0.821	0.786	0.750	0.714	0.821	0.741	0.750	0.786	0.857	0.857	0.821	0.821	0.893	0.857	0.229	0.679	0.714	0.714	0.821	0.821	0.821	0.815	0.815	0.778	0.857	0.786	0.786	0.714	0.750	0.821	0.821	0.167	0.815	0.704	0.821	0.714	0.679	1.000
**157**	0.857	0.821	0.893	0.893	0.786	0.857	0.679	0.821	0.679	0.857	0.821	0.607	0.714	0.607	0.679	0.821	0.750	0.643	0.679	0.714	0.786	0.786	0.216	0.821	0.786	0.679	0.893	0.679	0.714	0.750	0.857	0.750	0.786	0.607	0.857	0.893	0.750	0.821	0.857	0.786	0.679	0.679	0.750	0.778	0.786	0.786	0.821	0.893	0.786	0.857	0.857	0.857	0.229	0.714	0.821	0.786	0.857	0.857	0.893	0.852	0.778	0.704	0.857	0.857	0.786	0.786	0.786	0.929	0.857	0.167	0.778	0.778	0.857	0.821	0.714	0.893	1.000
**158**	0.679	0.571	0.679	0.714	0.679	0.643	0.643	0.714	0.714	0.679	0.714	0.643	0.679	0.679	0.714	0.679	0.821	0.786	1.000	0.679	0.571	0.679	0.270	0.643	0.679	0.607	0.607	1.000	0.857	0.643	0.679	0.750	0.643	0.607	0.714	0.714	0.643	0.571	0.714	0.607	0.679	0.643	0.679	0.667	0.750	0.643	0.786	0.750	0.821	0.714	0.679	0.714	0.229	0.714	0.607	0.607	0.714	0.679	0.643	0.667	0.815	0.667	0.679	0.607	0.679	0.607	0.643	0.643	0.679	0.167	0.741	0.704	0.714	0.643	0.750	0.714	0.679	1.000
**159**	0.714	0.679	0.750	0.679	0.714	0.679	0.607	0.821	0.643	0.750	0.750	0.571	0.643	0.571	0.643	0.750	0.714	0.607	0.786	0.643	0.714	0.786	0.216	0.643	0.750	0.714	0.750	0.786	0.679	0.643	0.821	0.679	0.643	0.643	0.679	0.786	0.679	0.714	0.714	0.750	0.786	0.750	0.679	0.704	0.821	0.750	0.750	0.750	0.786	0.714	0.750	0.786	0.229	0.679	0.571	0.679	0.750	0.679	0.679	0.667	0.741	0.778	0.714	0.714	0.786	0.714	0.750	0.750	0.714	0.167	0.778	0.704	0.679	0.750	0.679	0.786	0.750	0.786	1.000
**160**	0.964	0.929	0.929	0.857	0.714	0.786	0.679	0.786	0.750	0.857	0.929	0.643	0.679	0.643	0.750	0.821	0.714	0.643	0.643	0.679	0.750	0.714	0.162	0.857	0.857	0.750	0.929	0.643	0.714	0.714	0.750	0.786	0.750	0.643	0.893	0.786	0.750	0.786	0.786	0.786	0.643	0.679	0.714	0.778	0.714	0.786	0.786	0.786	0.750	0.821	0.786	0.821	0.229	0.714	0.857	0.821	0.750	0.821	0.893	0.815	0.741	0.704	0.964	0.964	0.893	0.857	0.821	0.857	0.750	0.167	0.741	0.741	0.893	0.750	0.821	0.821	0.893	0.643	0.679	1.000
**161**	0.964	0.929	0.929	0.857	0.714	0.786	0.679	0.786	0.750	0.857	0.929	0.643	0.679	0.643	0.750	0.821	0.714	0.643	0.643	0.679	0.750	0.714	0.162	0.857	0.857	0.750	0.929	0.643	0.714	0.714	0.750	0.786	0.750	0.643	0.893	0.786	0.750	0.786	0.786	0.786	0.643	0.679	0.714	0.778	0.714	0.786	0.786	0.786	0.750	0.821	0.786	0.821	0.229	0.714	0.857	0.821	0.750	0.821	0.893	0.815	0.741	0.704	0.964	0.964	0.893	0.857	0.821	0.857	0.750	0.167	0.741	0.741	0.893	0.750	0.821	0.821	0.893	0.643	0.679	1.000	1.000
**162**	0.786	0.821	0.821	0.786	0.643	0.714	0.571	0.679	0.607	0.750	0.750	0.643	0.571	0.571	0.607	0.893	0.714	0.607	0.607	0.571	0.750	0.714	0.162	0.821	0.750	0.643	0.893	0.607	0.679	0.571	0.714	0.679	0.679	0.607	0.786	0.679	0.679	0.750	0.679	0.750	0.643	0.679	0.679	0.630	0.714	0.750	0.714	0.714	0.679	0.750	0.714	0.750	0.229	0.679	0.714	0.786	0.679	0.750	0.821	0.667	0.630	0.741	0.786	0.857	0.786	0.750	0.821	0.857	0.714	0.167	0.667	0.593	0.750	0.714	0.750	0.750	0.786	0.607	0.643	0.821	0.821	1.000
**163**	0.786	0.750	0.821	0.821	0.786	0.786	0.643	0.893	0.679	0.821	0.821	0.500	0.679	0.571	0.679	0.786	0.821	0.714	0.714	0.679	0.786	0.786	0.216	0.786	0.750	0.643	0.821	0.714	0.786	0.679	0.786	0.750	0.714	0.643	0.857	0.821	0.786	0.750	0.786	0.750	0.750	0.750	0.714	0.741	0.821	0.786	0.821	0.857	0.857	0.821	0.786	0.893	0.229	0.679	0.714	0.786	0.821	0.786	0.857	0.704	0.704	0.704	0.786	0.786	0.786	0.750	0.857	0.857	0.821	0.167	0.704	0.741	0.750	0.786	0.857	0.786	0.857	0.714	0.786	0.821	0.821	0.750	1.000
**164**	0.679	0.643	0.679	0.750	0.643	0.607	0.607	0.714	0.643	0.679	0.714	0.571	0.643	0.607	0.643	0.750	0.964	0.929	0.857	0.643	0.607	0.643	0.216	0.714	0.750	0.643	0.679	0.857	1.000	0.607	0.643	0.714	0.607	0.571	0.750	0.679	0.643	0.643	0.750	0.607	0.607	0.643	0.679	0.704	0.714	0.714	0.750	0.821	0.786	0.679	0.679	0.750	0.229	0.714	0.643	0.679	0.679	0.643	0.714	0.630	0.704	0.667	0.679	0.679	0.679	0.679	0.714	0.679	0.714	0.167	0.704	0.704	0.679	0.679	0.821	0.714	0.714	0.857	0.679	0.714	0.714	0.679	0.786	1.000
**165**	0.679	0.571	0.643	0.607	0.750	0.643	0.750	0.643	0.786	0.571	0.714	0.893	0.750	1.000	0.786	0.643	0.607	0.536	0.679	0.750	0.500	0.607	0.216	0.643	0.643	0.679	0.571	0.679	0.607	0.643	0.679	0.750	0.714	0.679	0.679	0.643	0.571	0.607	0.679	0.571	0.679	0.607	0.571	0.481	0.607	0.464	0.607	0.571	0.643	0.607	0.607	0.571	0.229	0.643	0.786	0.500	0.750	0.679	0.571	0.667	0.630	0.593	0.679	0.607	0.679	0.607	0.607	0.571	0.536	0.167	0.556	0.667	0.750	0.607	0.679	0.607	0.607	0.679	0.571	0.643	0.643	0.571	0.571	0.607	1.000
**166**	0.786	0.750	0.821	0.750	0.714	0.714	0.643	0.821	0.679	0.893	0.750	0.679	0.679	0.607	0.679	0.786	0.679	0.571	0.643	0.679	0.679	0.821	0.162	0.679	0.679	0.714	0.821	0.643	0.643	0.714	0.821	0.643	0.750	0.714	0.679	0.821	0.786	0.786	0.714	0.964	0.714	0.786	0.679	0.630	0.750	0.750	0.786	0.679	0.714	0.786	0.714	0.786	0.229	0.679	0.679	0.821	0.679	0.821	0.750	0.778	0.704	0.741	0.786	0.786	0.786	0.679	0.714	0.786	0.643	0.167	0.741	0.667	0.714	0.750	0.679	0.750	0.750	0.643	0.714	0.750	0.750	0.786	0.714	0.643	0.607	1.000
**167**	0.821	0.857	0.786	0.714	0.571	0.643	0.607	0.679	0.643	0.786	0.786	0.536	0.607	0.536	0.643	0.714	0.643	0.571	0.571	0.607	0.679	0.607	0.162	0.714	0.786	0.643	0.786	0.571	0.643	0.643	0.643	0.643	0.607	0.571	0.750	0.714	0.643	0.643	0.679	0.714	0.500	0.571	0.714	0.889	0.643	0.679	0.679	0.714	0.643	0.714	0.679	0.714	0.229	0.643	0.750	0.750	0.643	0.679	0.750	0.778	0.704	0.667	0.821	0.821	0.750	0.857	0.679	0.714	0.750	0.167	0.741	0.667	0.786	0.714	0.714	0.714	0.786	0.571	0.607	0.857	0.857	0.679	0.714	0.643	0.536	0.679	1.000
**168**	0.786	0.821	0.821	0.929	0.786	0.821	0.643	0.750	0.714	0.786	0.750	0.679	0.679	0.571	0.714	0.786	0.750	0.643	0.679	0.679	0.857	0.857	0.216	0.857	0.821	0.679	0.893	0.679	0.714	0.643	0.821	0.786	0.786	0.679	0.821	0.786	0.714	0.893	0.821	0.786	0.786	0.750	0.714	0.704	0.786	0.857	0.857	0.893	0.821	0.821	0.821	0.821	0.229	0.786	0.750	0.821	0.786	0.821	0.857	0.741	0.778	0.704	0.786	0.857	0.786	0.750	0.857	0.964	0.821	0.167	0.815	0.704	0.786	0.786	0.714	0.857	0.893	0.679	0.750	0.821	0.821	0.821	0.821	0.714	0.571	0.750	0.679	1.000
**169**	0.821	0.857	0.786	0.714	0.643	0.643	0.571	0.714	0.607	0.714	0.857	0.607	0.571	0.607	0.607	0.857	0.679	0.607	0.607	0.571	0.679	0.643	0.162	0.714	0.929	0.786	0.857	0.607	0.679	0.607	0.786	0.714	0.607	0.643	0.821	0.679	0.607	0.714	0.750	0.714	0.643	0.679	0.607	0.704	0.750	0.714	0.643	0.679	0.679	0.679	0.679	0.679	0.229	0.679	0.750	0.750	0.714	0.679	0.750	0.704	0.630	0.815	0.821	0.893	0.893	0.929	0.821	0.786	0.643	0.167	0.704	0.704	0.786	0.786	0.750	0.714	0.786	0.607	0.714	0.857	0.857	0.821	0.750	0.679	0.607	0.679	0.786	0.750	1.000
**170**	0.679	0.714	0.714	0.786	0.786	0.714	0.607	0.750	0.607	0.750	0.714	0.571	0.643	0.571	0.607	0.821	0.750	0.643	0.679	0.643	0.750	0.821	0.216	0.750	0.786	0.714	0.786	0.679	0.714	0.607	0.821	0.714	0.714	0.750	0.786	0.750	0.643	0.750	0.786	0.750	0.714	0.750	0.714	0.667	0.821	0.750	0.750	0.821	0.786	0.750	0.786	0.750	0.229	0.714	0.679	0.786	0.786	0.714	0.750	0.667	0.741	0.815	0.679	0.750	0.750	0.750	0.857	0.857	0.857	0.167	0.741	0.741	0.714	0.857	0.750	0.786	0.821	0.679	0.786	0.714	0.714	0.750	0.821	0.714	0.571	0.714	0.679	0.857	0.750	1.000
