# Supplementary material for: Assessment of Wild Rocket (Diplotaxis tenuifolia (L.) DC.) Germplasm Accessions by NGS Identified SSR and SNP Markers
Source: Plants (Basel). 2022 Dec 12;11(24):3482. doi: 10.3390/plants11243482 (PMC9783343; doi:10.3390/plants11243482)
Supplement: Supplementary file 1 [file plants-11-03482-s001.zip › Table S4 .pdf]

Table S4. Molecular fingerprints of the 90 accessions (SSR and SNP/CAPS markers)

ACCESSIONS	MT317577.1	MT317527.1	MT317610.1	MT317537.1	MT317823.1	DiploTSNP.045	DiploTSNP.062	DiploTSNP.090	DiploTSNP.138	DiploTSNP.213	DiploTSNP.220	DiploTSNP.270	DiploTSNP.290	DiploTSNP.448
**1**	248;248	179;181	157;157	163;175	182;184	Y/Y	Y/Y	Y/Y	Y/Y	Y/Y	Y/Y	N/N	Y/N	N/N
**3**	242;248	179;181	157;157	163;175	182;184	Y/Y	Y/Y	Y/Y	Y/Y	Y/N	Y/N	N/N	Y/Y	N/N
**4**	242;248	179;181	157;157	163;175	182;184	Y/Y	Y/Y	Y/Y	Y/Y	Y/Y	Y/Y	N/N	Y/N	N/N
**5**	242;252	173;181	157;157	163;175	184;188	Y/Y	Y/Y	Y/Y	Y/Y	Y/N	Y/Y	N/N	Y/N	N/N
**6**	242;242	173;181	157;157	163;175	182;184	Y/Y	Y/Y	Y/N	Y/N	Y/Y	Y/Y	N/N	N/N	N/N
**7**	242;242	173;181	157;157	163;175	182;184	Y/N	Y/Y	Y/Y	Y/N	Y/Y	Y/Y	N/N	Y/N	N/N
**8**	244;248	173;179	157;157	163;171	182;184	Y/N	Y/Y	Y/N	Y/N	Y/Y	Y/Y	N/N	Y/N	N/N
**9**	242;242	179;181	157;157	163;171	182;184	Y/Y	Y/Y	Y/N	Y/Y	Y/Y	Y/Y	N/N	N/N	N/N
**10**	248;248	173;181	157;157	163;171	186;188	Y/Y	Y/Y	Y/N	N/N	Y/Y	Y/Y	N/N	Y/N	N/N
**12**	242;252	173;179	157;157	163;171	182;184	Y/Y	Y/Y	Y/Y	Y/Y	Y/Y	Y/Y	N/N	Y/N	N/N
**15**	248;248	179;181	157;157	163;175	184;188	Y/Y	Y/Y	Y/Y	Y/Y	Y/Y	Y/Y	N/N	N/N	N/N
**16**	248;248	173;181	157;157	163;175	156;188	Y/Y	Y/Y	Y/N	N/N	Y/Y	Y/N	N/N	Y/N	Y/N
**17**	244;244	173;181	157;157	163;171	156;184	Y/Y	Y/Y	Y/N	Y/N	Y/Y	Y/Y	N/N	Y/N	N/N
**18**	248;248	173;173	157;157	163;175	156;156	Y/Y	Y/Y	Y/Y	N/N	Y/Y	Y/Y	N/N	N/N	Y/N
**19**	248;248	173;181	157;157	163;171	156;188	Y/Y	Y/Y	Y/N	Y/N	Y/Y	Y/Y	N/N	Y/N	N/N
**20**	242;248	177;179	157;157	163;175	182;184	Y/Y	Y/Y	Y/Y	Y/Y	Y/N	Y/N	N/N	N/N	Y/N
**21**	238;242	161;167	157;157	163;171	184;188	Y/Y	Y/Y	Y/Y	Y/Y	Y/N	Y/Y	N/N	N/N	Y/N
**22**	238;238	161;167	157;157	163;171	184;188	Y/N	Y/Y	Y/Y	Y/Y	Y/N	Y/Y	Y/N	N/N	Y/N
**23**	238;238	171;173	157;157	163;175	186;188	Y/Y	Y/Y	Y/Y	Y/Y	Y/Y	Y/Y	N/N	N/N	Y/N
**24**	244;244	173;181	157;157	163;171	156;184	Y/Y	Y/Y	Y/Y	Y/N	Y/Y	Y/Y	N/N	Y/N	N/N
**26**	242;242	179;181	157;157	163;175	211;213	Y/Y	Y/Y	Y/Y	Y/Y	N/N	Y/N	N/N	Y/N	N/N
**27**	242;248	173;181	157;157	163;171	182;184	Y/Y	Y/Y	Y/N	Y/Y	Y/Y	Y/N	N/N	Y/N	N/N
**29**	X	173;170	135;157	167;171	X	Y/Y	X	X	X	X	X	X	X	X
**32**	242;248	177;181	157;157	163;175	184;188	Y/Y	Y/Y	Y/Y	Y/Y	Y/N	Y/Y	N/N	Y/N	N/N
**35**	242;242	179;179	157;157	163;175	184;188	Y/Y	Y/Y	Y/Y	Y/Y	Y/N	Y/N	N/N	N/N	N/N
**36**	248;248	167;179	157;157	163;175	182;184	Y/Y	Y/Y	Y/N	Y/Y	Y/Y	Y/N	Y/N	N/N	N/N
**37**	242;248	179;181	157;157	163;175	182;184	Y/Y	Y/Y	Y/Y	Y/Y	Y/N	Y/N	N/N	Y/N	N/N
**38**	238;238	171;173	157;157	163;171	186;188	Y/Y	Y/Y	Y/Y	Y/Y	Y/Y	Y/Y	N/N	N/N	Y/N
**39**	238;242	167;167	157;157	163;171	184;188	Y/Y	Y/Y	Y/Y	Y/Y	Y/N	Y/Y	N/N	N/N	N/N
**40**	244;244	173;179	157;157	163;171	182;184	Y/Y	Y/Y	Y/N	Y/N	Y/Y	Y/Y	N/N	Y/N	N/N
**41**	238;242	173;179	157;157	163;175	182;184	Y/Y	Y/Y	Y/N	Y/Y	Y/Y	Y/N	N/N	N/N	N/N
**42**	246;248	173;181	157;157	163;175	184;188	Y/Y	Y/Y	Y/Y	Y/Y	Y/Y	Y/Y	N/N	N/N	N/N
**43**	242;246	173;181	157;157	163;175	182;184	Y/Y	Y/Y	Y/N	Y/Y	Y/Y	Y/Y	N/N	Y/N	N/N
**44**	240;248	173;181	157;157	163;175	182;184	Y/Y	Y/Y	Y/N	Y/Y	Y/Y	Y/N	Y/N	N/N	N/N
**45**	246;248	173;181	157;157	163;175	182;184	Y/Y	Y/Y	Y/Y	Y/Y	Y/N	Y/Y	N/N	N/N	N/N
**46**	242;248	173;179	157;157	163;175	182;184	Y/Y	Y/Y	Y/N	Y/Y	Y/Y	Y/Y	N/N	Y/N	N/N
**47**	242;246	161;181	157;157	163;171	182;184	Y/Y	Y/Y	Y/N	Y/Y	Y/Y	Y/Y	Y/N	Y/N	N/N
**48**	242;242	179;181	157;157	163;175	184;188	Y/Y	Y/Y	Y/N	Y/N	Y/N	Y/N	N/N	N/N	N/N
**49**	242;246	173;179	157;175	163;175	184;188	Y/Y	Y/Y	Y/N	Y/Y	Y/N	Y/Y	N/N	N/N	N/N
**51**	242;252	179;181	157;173	163;177	182;184	Y/Y	Y/Y	Y/N	Y/Y	Y/Y	Y/N	N/N	Y/N	N/N
**52**	242;242	171;181	157;157	163;175	186;188	Y/Y	Y/Y	Y/N	N/N	Y/Y	Y/N	N/N	N/N	N/N
**54**	242;246	161;181	157;157	163;175	184;213	Y/Y	Y/Y	Y/N	Y/Y	Y/Y	Y/N	N/N	N/N	N/N
**55**	238;242	161;177	157;157	163;173	156;184	Y/Y	Y/Y	Y/Y	Y/Y	Y/Y	Y/Y	N/N	Y/Y	N/N
**56**	X	179;179	157;177	163;171	184;186	Y/Y	Y/Y	Y/Y	Y/Y	N/N	Y/Y	N/N	Y/Y	N/N
**57**	242;244	161;173	157;157	163;171	182;184	Y/Y	Y/Y	Y/Y	Y/Y	Y/Y	Y/N	N/N	N/N	N/N

ACCESSIONS	MT317577.1	MT317527.1	MT317610.1	MT317537.1	MT317823.1	DiploTSNP.045	DiploTSNP.062	DiploTSNP.090	DiploTSNP.138	DiploTSNP.213	DiploTSNP.220	DiploTSNP.270	DiploTSNP.290	DiploTSNP.448
**58**	242;246	179;181	157;157	163;175	184;188	Y/Y	Y/Y	Y/Y	Y/Y	Y/N	Y/N	Y/N	Y/N	N/N
**59**	242;252	173;181	157;157	163;171	184;188	Y/Y	Y/Y	Y/Y	Y/Y	Y/Y	Y/Y	N/N	Y/N	N/N
**60**	242;242	167;173	157;175	163;171	184;188	Y/Y	Y/Y	Y/Y	Y/Y	Y/N	Y/Y	N/N	Y/N	N/N
**61**	242;246	173;181	157;157	163;171	184;188	Y/Y	Y/Y	Y/Y	Y/Y	Y/Y	Y/Y	N/N	N/N	N/N
**63**	242;252	173;181	157;157	163;173	182;184	Y/Y	Y/Y	Y/Y	Y/Y	Y/Y	Y/Y	N/N	Y/N	N/N
**64**	242;242	161;173	157;157	163;175	182;184	Y/Y	Y/Y	Y/Y	Y/Y	Y/Y	Y/Y	N/N	Y/N	N/N
**65**	242;242	167;181	157;175	163;171	182;184	Y/Y	Y/Y	Y/Y	Y/Y	Y/Y	Y/Y	N/N	Y/N	N/N
**68**	X	170;170	157;157	168;300	X	Y/Y	X	X	X	X	X	X	X	X
**69**	238;248	171;173	157;157	163;171	184;188	Y/Y	Y/Y	Y/N	Y/N	Y/N	Y/N	N/N	Y/N	N/N
**72**	246;248	173;173	157;157	163;175	182;184	Y/Y	Y/Y	Y/N	Y/N	Y/N	Y/Y	N/N	Y/N	N/N
**73**	250;252	161;181	157;157	163;175	182;184	Y/Y	Y/Y	Y/Y	Y/Y	Y/N	Y/N	N/N	Y/N	N/N
**74**	242;242	173;181	157;157	163;175	182;184	Y/Y	Y/Y	Y/Y	Y/N	Y/Y	Y/Y	N/N	N/N	N/N
**81**	242;246	173;181	157;157	163;175	182;184	Y/Y	Y/Y	Y/N	Y/Y	Y/Y	Y/Y	N/N	Y/N	N/N
**84**	242;244	161;181	157;157	163;175	182;184	Y/Y	Y/Y	Y/Y	Y/Y	Y/N	Y/Y	N/N	Y/N	N/N
**91**	X	173;179	157;157	163;175	182;184	Y/Y	Y/Y	Y/N	Y/Y	Y/Y	Y/Y	N/N	Y/Y	N/N
**92**	X	161;173	157;157	163;173	186;188	Y/Y	Y/Y	Y/Y	Y/Y	Y/Y	Y/Y	N/N	Y/N	N/N
**97**	X	177;179	157;157	163;173	156;184	Y/Y	Y/Y	Y/Y	Y/Y	Y/Y	N/N	N/N	N/N	N/N
**98**	242;248	179;181	157;157	163;175	182;184	Y/Y	Y/Y	Y/Y	Y/Y	Y/Y	Y/N	N/N	Y/N	N/N
**99**	248;248	179;181	157;157	163;175	182;184	Y/Y	Y/Y	Y/Y	Y/Y	Y/N	Y/N	N/N	Y/N	N/N
**100**	248;248	173;181	157;175	163;175	182;184	Y/Y	Y/Y	Y/Y	Y/Y	Y/Y	Y/N	N/N	N/N	N/N
**101**	248;248	177;179	157;157	163;175	182;184	Y/Y	Y/Y	Y/Y	Y/Y	N/N	Y/N	N/N	N/N	N/N
**102**	242;248	179;181	157;157	163;175	182;184	Y/Y	Y/Y	Y/Y	Y/Y	Y/N	Y/N	N/N	Y/N	N/N
**103**	242;242	173;181	157;171	163;175	182;184	Y/Y	Y/Y	Y/Y	Y/Y	Y/N	Y/N	N/N	Y/N	N/N
**104**	242;242	173;177	157;157	163;171	182;184	Y/Y	Y/Y	Y/Y	Y/Y	Y/N	Y/N	N/N	Y/N	N/N
**105**	X	162;170	135;157	172;300	X	Y/Y	X	X	X	X	X	X	X	X
**134**	X	173;179	157;157	163;171	184;188	Y/Y	Y/Y	Y/Y	Y/Y	Y/Y	Y/N	N/N	Y/Y	N/N
**146**	X	173;179	157;157	163;177	211;213	Y/Y	Y/Y	Y/N	Y/Y	Y/N	Y/Y	N/N	N/N	N/N
**152**	238;248	173;181	157;173	163;175	182;184	Y/Y	Y/Y	Y/Y	Y/Y	Y/Y	Y/Y	N/N	Y/N	N/N
**154**	240;242	161;173	157;157	163;171	182;184	Y/Y	Y/Y	Y/Y	Y/N	Y/N	Y/N	N/N	Y/N	N/N
**155**	248;248	179;181	157;173	163;171	182;184	Y/Y	Y/Y	Y/Y	Y/Y	Y/N	Y/Y	N/N	N/N	Y/N
**156**	242;242	177;179	157;171	163;175	184;188	Y/Y	Y/Y	Y/Y	Y/Y	Y/Y	Y/Y	N/N	Y/N	N/N
**157**	242;242	173;179	157;157	163;175	182;184	Y/Y	Y/Y	Y/Y	Y/Y	Y/N	Y/Y	N/N	Y/N	N/N
**158**	238;238	171;173	157;157	163;171	186;188	Y/Y	Y/Y	Y/Y	Y/Y	Y/Y	Y/Y	N/N	N/N	Y/N
**159**	242;242	171;179	157;157	163;171	186;186	Y/Y	Y/Y	Y/Y	Y/Y	Y/Y	Y/N	N/N	N/N	N/N
**160**	248;248	179;181	157;157	163;175	182;184	Y/Y	Y/Y	Y/Y	Y/Y	Y/N	Y/Y	N/N	Y/N	N/N
**161**	248;248	179;181	157;157	163;175	182;184	Y/Y	Y/Y	Y/Y	Y/Y	Y/N	Y/Y	N/N	Y/N	N/N
**162**	242;248	177;181	157;157	163;175	182;184	Y/Y	Y/Y	Y/Y	Y/Y	Y/N	N/N	N/N	Y/N	N/N
**163**	242;242	179;181	157;157	163;171	182;184	Y/Y	Y/Y	Y/Y	Y/Y	Y/N	Y/Y	N/N	N/N	N/N
**164**	238;238	167;167	157;157	163;171	186;188	Y/Y	Y/Y	Y/Y	Y/Y	Y/N	Y/Y	N/N	N/N	N/N
**165**	248;248	173;173	157;157	163;175	156;188	Y/Y	Y/Y	Y/N	N/N	Y/Y	Y/Y	N/N	N/N	N/N
**166**	242;252	179;181	157;157	163;177	182;184	Y/Y	Y/Y	Y/N	Y/Y	Y/Y	Y/N	N/N	Y/N	N/N
**167**	248;248	179;181	157;157	163;175	182;184	Y/Y	Y/Y	Y/Y	Y/Y	N/N	Y/Y	N/N	Y/Y	N/N
**168**	242;242	173;181	157;157	163;175	184;188	Y/Y	Y/Y	Y/Y	Y/Y	Y/N	Y/N	N/N	Y/N	N/N
**169**	248;248	177;179	157;157	163;175	182;184	Y/Y	Y/Y	Y/Y	Y/Y	Y/N	N/N	N/N	N/N	N/N
**170**	242;246	173;177	157;157	163;177	182;184	Y/Y	Y/Y	Y/Y	Y/Y	Y/N	Y/N	N/N	N/N	N/N
